# The Interplay between Error, Total Variation, Alpha-Entropy and Guessing: Fano and Pinsker Direct and Reverse Inequalities §

**DOI:** 10.3390/e25070978

**Published:** 2023-06-25

**Authors:** Olivier Rioul

**Affiliations:** LTCI, Télécom Paris, Institut Polytechnique de Paris, 91120 Palaiseau, France; olivier.rioul@telecom-paris.fr

**Keywords:** entropy, Rényi entropy, guessing entropy, guessing moments, total variation distance, error probability, data processing inequality, majorization, Schur concavity, Fano inequality, Pinsker inequality

## Abstract

Using majorization theory via “Robin Hood” elementary operations, optimal lower and upper bounds are derived on Rényi and guessing entropies with respect to either error probability (yielding reverse-Fano and Fano inequalities) or total variation distance to the uniform (yielding reverse-Pinsker and Pinsker inequalities). This gives a general picture of how the notion of randomness can be measured in many areas of computer science.

## 1. Introduction

In many areas of science, it is of primary importance to assess the “randomness” of a certain random variable *X*. That variable could represent, for example, a cryptographic key, a signature, some sensitive data, or any type of intended secret. For simplicity, we assume that *X* is an *M*-ary discrete random variable, taking values in a finite alphabet X of size *M*, with known probability distribution $p=(p_{1},p_{2},\ldots ,p_{M})$ (in short, X∼p).

Depending on the application, many different criteria can be used to evaluate randomness. Some are information-theoretic, others are related to detection/estimation theory or to hypothesis testing. We review the most common ones in the following subsections.

### 1.1. Entropy

A “sufficiently random” *X* is often described as “entropic” in the literature. The usual notion of entropy is the Shannon entropy [1]

(1) $$H\left(X\right)=H\left(p\right)≜\sum_{k}p_{k}\cdot \log\frac{1}{p_{k}},$$

which is classically thought of as a measure of “uncertainty”. It has, however, an operational definition in the fields of data compression or source coding. The problem is to find the binary description of *X* with the shortest average description length or “coding rate”.

Note that the base of the logarithm is not specified in (1). Similar to all information-theoretic quantities, the choice of the base determines the unit of information. Logarithms of base 2 give binary units (bits) or Shannons (Sh). Logarithms of base 10 give decimal units (dits) or Hartleys. Natural logarithms (base *e*) give natural units (nats).

This compression problem can be seen as equivalent to a “game of 20 questions” § 5.7.1 in [2], where a binary codeword for *X* is identified as a sequence of answers to yes–no questions about *X* that uniquely identifies it. There is no limitation on the type of questions asked, except that they must be answered by yes (1) or no (0). The goal of the game is to minimize the average number of questions, which is equal to the coding rate. It is well known, since Shannon [1], that the entropy H(X) is a lower bound on the coding rate that can be achieved asymptotically for repeated descriptions.

In this perspective, entropy is a natural measure of efficient (lossless) compression rate. A highly random variable (with high entropy) cannot be compressed too much without losing information: “random” means “hard to compress”.

### 1.2. Guessing Entropy

Another perspective arises in cryptography when one wants to guess a secret key. The situation is similar to the “game of 20 questions” of the preceding subsection. The difference is that the only possibility is to actually try out one possible key hypothesis at a time. In other words, yes–no questions are restricted to be of the form “is *X* equal to *x*?” until the correct value has been found. The optimal strategy that minimizes the average number of questions is to guess the values of *X* in order of decreasing probabilities: first, the value with maximum probability $p_{\left(1\right)}$, then the second maximum $p_{\left(2\right)}$, and so on. The corresponding minimum average number of guesses is the guessing entropy [3] (also known as “guesswork” [4]):

(2) $$G\left(X\right)=G\left(p\right)≜\sum_{k}p_{\left(k\right)}\cdot k.$$

Massey [3] has shown that the guessing entropy *G* is exponentially increasing as entropy *H* increases. A recent improved inequality is [5,6] $G>\frac{\exp H}{e}+\frac{1}{2}$. It is sometimes convenient to use logG instead of *G*, to express it in the same logarithmic unit of information as entropy *H*.

In this perspective, a highly random variable (with high guessing entropy) cannot be guessed rapidly: “random” means “hard to guess”.

### 1.3. Coincidence or Collision

Another perspective is to view *X* as a (publicly available) “identifier”, “fingerprint” or “signature” obtained by a randomized algorithm from some sensitive data. In such a scheme, to prevent “collision attacks”, it is important to ensure that *X* is “unique” in the sense that there is only a small chance that another independent $X^{\prime }$ obtained by the same randomized algorithm coincides with *X*. Since *X* and $X^{\prime }$ are i.i.d., the “index of coincidence” $P\left(X=X^{\prime }\right)=\sum_{k}p_{k}^{2}$ should be as small as possible, that is, the complementary quantity (sometimes called quadratic entropy [7]):

(3) $$R_{2}\left(X\right)=R_{2}\left(p\right)≜P\left(X\neq X^{\prime }\right)=1-\sum_{k}p_{k}^{2},$$

should be as large as possible. In the context of hash functions, this is called “universality” (Chapter 8 in [8]). The corresponding logarithmic measure is known as the collision entropy (Rényi entropy [9] of order 2, also known as quadratic entropy [10]):

(4) $$H_{2}\left(X\right)=H_{2}\left(p\right)≜\log\frac{1}{1-R_{2}\left(X\right)}=\log\frac{1}{\sum_{k}p_{k}^{2}}$$

which should also be as large as possible. By concavity of the logarithm, $\sum_{k}p_{k}\log p_{k}\leq \log\sum_{k}p_{k}^{2}$, that is, $H\geq H_{2}$; hence, high collision entropy implies high entropy.

In this perspective, a highly random variable (with high collision entropy) cannot be found easily by coincidence: “random” means “unique” or “hard to collide”.

### 1.4. Estimation Error

In estimation or detection theory, one observes some disclosed data which may depend on *X* and tries to estimate *X* from the observation. The best estimator $\hat{x}$ minimizes the probability of error, $P\left(X\neq \hat{x}\right)=1-P\left(X=\hat{x}\right)$. Therefore, given the observation, the best estimation is the value *x* with highest probability $p_{\left(1\right)}$, and the minimum probability of error is written:

(5) $$P_{e}\left(X\right)=P_{e}\left(p\right)≜1-\max p=1-p_{\left(1\right)}.$$

If *X* is meant to be kept secret, then this probability of error should be as large as possible. The corresponding logarithmic measure is known as the min-entropy:

(6) $$H_{\infty }\left(X\right)=H_{\infty }\left(p\right)≜\log\frac{1}{1-P_{e}\left(X\right)}=\log\frac{1}{p_{\left(1\right)}}$$

which should also be as large as possible. It is easily seen that $H\geq H_{2}\geq H_{\infty }$; hence, high min-entropy implies high entropy in all the previous senses.

In this perspective, a highly random variable (with high min-entropy) cannot be efficiently estimated: “random” means “hard to estimate” or “hard to detect”.

**Figure 1:**
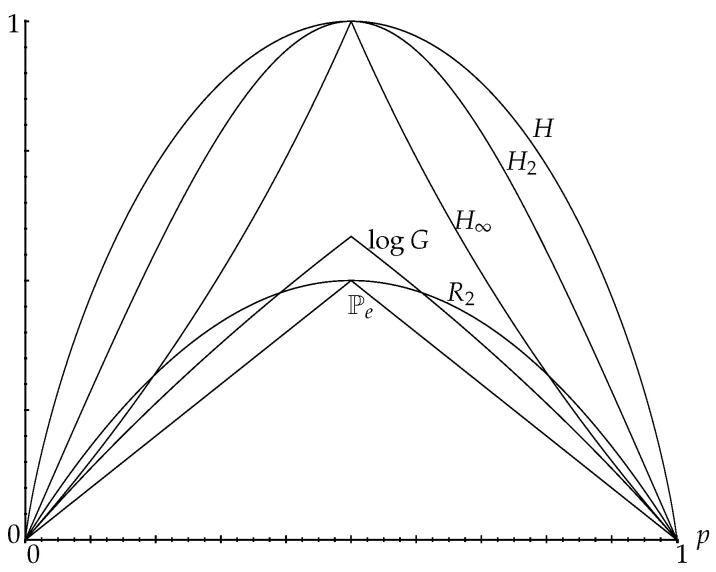
Various randomness measures (in bits) for a binary distribution (p,1−p) as a function of *p*.

Figure 1 illustrates various randomness measures for a binary distribution.

### 1.5. Some Generalizations

One can generalize the above concepts in multiple ways. We only mention a few.

The α-entropy, or Rényi entropy of order α>0, is defined as follows [9]:

(7) $$H_{\alpha }\left(X\right)=H_{\alpha }\left(p\right)≜\frac{1}{1-\alpha }\log\sum_{k}p_{k}^{\alpha }=\frac{\alpha }{1-\alpha }\log{∥p∥}_{\alpha }$$

where ${∥\cdot ∥}_{\alpha }$ is the “α-norm” (strictly speaking, ${∥\cdot ∥}_{\alpha }$ is a norm only when α≥1). The Shannon entropy $H=H_{1}$ is recovered in the limiting case α→1, the collision entropy $H_{2}$ is recovered in the case α=2, and the min-entropy $H_{\infty }$ is recovered in the limiting case α→∞.

The ρ-guessing entropy, or guessing moment [11] of order ρ>0, is defined as the minimum ρth-order moment of the number of guesses needed to find *X*. The same optimal strategy as for the guessing entropy yields the following:

(8) $$G_{\rho }\left(X\right)=G_{\rho }\left(p\right)≜\sum_{k}p_{\left(k\right)}\cdot k^{\rho },$$

which generalizes $G=G_{1}$ for ρ≠1. Arikan [11] has shown that $\log G_{\rho }$ behaves asymptotically as $\rho H_{\frac{1}{1+\rho }}$. In particular, logG behaves asymptotically as the ½-entropy $H_{\frac{1}{2}}$.

In some cryptographic scenarios, one has the ability to estimate or guess *X* in a given maximum number *m* of tries. The corresponding error probability takes the form $P(X\neq {\hat{x}}_{1},X\neq {\hat{x}}_{2},\ldots ,X\neq {\hat{x}}_{m})$. The same optimal strategy as for guessing entropy $G_{\rho }$ yields an error probability of order *m*:

(9) $$P_{e}^{m}\left(X\right)=P_{e}^{m}\left(p\right)≜1-p_{\left(1\right)}-p_{\left(2\right)}-\cdots -p_{\left(m\right)},$$

which generalizes $P_{e}=P_{e}^{1}$ for m>1.

One obtains similar randomness measures by replacing *p* with its “negation” $\bar{p}$, as explained in [12].

### 1.6. “Distances” to the Uniform

A fairly common convention is that, if we “draw at random” *X*, it is assumed that we sample it according to a uniform distribution unless otherwise explicitly indicated. Thus, the uniform distribution *u*, where all possible outcomes being equally likely—all *M* values have equal probability $u_{k}=\frac{1}{M}$ for all *k*—is considered as the ideal randomness.

From this viewpoint, a variable *X* with distribution *p* should be all the more “random” as *p* is “close to uniform”: randomness can be measured as some complementary “distance” from *p* to the uniform *u*, in the form, say, $d_{\max}-d\left(p,u\right)$, where “distance” *d* has maximum value $d_{\max}$. Such d(p,u) should not necessarily obey all axioms of a mathematical distance, but at least should be nonnegative and vanish only when p=u.

Many of the above entropic criteria fall into this category. For example:

(10) H(p)=logM−D(p∥u),

where $D\left(p∥q\right)=\sum_{k}p_{k}\log\frac{p_{k}}{q_{k}}$ denotes the (Kullback–Leibler) divergence (or “distance”). More generally:

(11) $$H_{\alpha }\left(p\right)=\log M-D_{\alpha }\left(p∥u\right),$$

where $D_{\alpha }\left(p∥q\right)=\frac{1}{\alpha -1}\log\sum_{k}p_{k}^{\alpha }q_{k}^{1-\alpha }$ denotes the (Rényi) α-divergence [13].

In the particular case α=2, since $\sum_{k}{\left(p_{k}-\frac{1}{M}\right)}^{2}=\sum_{k}p_{k}^{2}-\frac{1}{M}$, the complementary index of coincidence $R_{2}$—hence, the collision entropy $H_{2}$—is also related to the squared 2-norm distance to the uniform:

(12) $$R_{2}\left(p\right)=\left(1-\frac{1}{M}\right)-{∥p-u∥}_{2}^{2}.$$

It follows that the 2-norm distance is related to the 2-divergence by the formula $D_{2}\left(p∥u\right)={\log(1+M∥p-u∥}_{2}^{2})$ (see, e.g., Lemma 3 in [14]).

Similarly, in the particular case $\alpha =\frac{1}{2}$, one can write $H_{\frac{1}{2}}\left(p\right)=2\log\left(1+R_{\frac{1}{2}}\left(p\right)\right)$, where

(13) $$\begin{array}{cc} R_{\frac{1}{2}}\left(p\right) & =\sum_{k}\sqrt{p_{k}}-1 \end{array}$$

(14) $$\begin{array}{cc} \; & =\sqrt{M}\left(\left(1-\frac{1}{\sqrt{M}}\right)-\frac{1}{2}{∥\sqrt{p}-\sqrt{u}∥}_{2}^{2}\right) \end{array}$$

is a complementary quantity of the squared *Hellinger distance* $\frac{1}{2}{∥\sqrt{p}-\sqrt{u}∥}_{2}^{2}$, which is related to the $\frac{1}{2}$-divergence by the formula $D_{1/2}\left(p∥u\right)=-2\log(1-\frac{1}{2}∥\sqrt{p}-\sqrt{u}{∥}_{2}^{2})$.

Another important example is given next.

### 1.7. Statistical Distance to the Uniform

Suppose one wants to design a statistical experiment to know whether *X* follows either distribution *p* (null hypothesis $H_{0}$) or another distribution *q* (alternate hypothesis). Any statistical test takes the form “is X∈T?”: if yes, then accept $H_{0}$; otherwise, reject it. Type-I and type-II errors have total probability P(X∉T)+Q(X∈T), where P, Q are the probability measures corresponding to *p* and *q*, respectively. Clearly, if |P(X∈T)−Q(X∈T)| is small enough, the two hypotheses *p* and *q* are indistinguishable in the sense that decision errors have total probability arbitrarily close to 1.

The statistical (total variation) distance § 8.8 in [8] is defined as follows:

(15) $$\Delta \left(p,q\right)=\max_{T}\left|P\left(T\right)-Q\left(T\right)\right|=\frac{1}{2}{∥p-q∥}_{1},$$

where the $\frac{1}{2}$ factor is present to ensure that 0≤Δ(p,q)≤1. The maximum in the definition of the statistical distance:

(16) $$\Delta \left(p,q\right)=\max_{T}\left|P\left(T\right)-Q\left(T\right)\right|=P\left(T_{+}\right)-Q\left(T_{+}\right)$$

is attained for any event $T_{+}$, satisfying the following:

(17) $$\left\{p>q\right\}\subset T_{+}\subset \left\{p\geq q\right\}.$$

The statistical distance is particularly important from a hypothesis testing viewpoint, since, as we have just seen, a very small distance Δ(p,q) ensures that no statistical test can distinguish the two hypotheses *p* and *q*.

Following the discussion of the preceding subsection, we can define “statistical randomness” as the complementary value of the statistical distance Δ(p,u) between *p* and the uniform distribution *u*. Therefore, if q=u is uniform and letting $K=|T_{+}|$, then $\Delta \left(p,u\right)=P\left(T_{+}\right)-\frac{K}{M}$ has maximum value $1-\frac{1}{M}$ and statistical randomness can be defined as follows:

(18) $$R\left(X\right)=R\left(p\right)≜\left(1-\frac{1}{M}\right)-\Delta \left(p,u\right)=\left(1-\frac{1}{M}\right)-\frac{1}{2}{∥p-u∥}_{1}.$$

This is similar to (12), where half the 1-norm is used in place of the squared 2-norm.

From the hypothesis testing perspective, it follows that a high statistical randomness *R* ensures that no statistical test can effectively distinguish between the actual distribution and the uniform. This is, for example, the usual criterion used to evaluate randomness extractors in cryptology. Since equiprobable values are the least predictable, a highly random variable cannot be easily statistically predicted: “random” means “hard to predict”.

### 1.8. Conditional Versions

In many applications, the randomness of *X* is evaluated after observing some disclosed data or side information *Y*. The observed random variable *Y* can model any type of data and is not necessarily discrete. The conditional probability distribution of *X* having observed Y=y is denoted by $p_{X|y}$ to distinguish it from the unconditional distribution $p=p_{X}$ (without side information). By the law of total probability $P\left(X=x\right)=E_{y}P\left(X=x|Y=y\right)$, $p_{X}$ is recovered by averaging all conditional distributions:

(19) $$p_{X}=E_{y}p_{X|y},$$

where $E_{y}$ denotes the expectation operator over *Y*.

The “conditional randomness” of *X* given *Y* can then be defined as the average randomness measure of X|y over all possible observations, that is, the expectation over *Y* of all randomness measures of X|Y=y. For example, Shannon’s conditional entropy or equivocation [1] is given by the following:

(20) $$H\left(X|Y\right)≜E_{y}H\left(X|y\right)=E_{y}H\left(p_{X|y}\right).$$

Similarly:

(21) $$G\left(X|Y\right)≜E_{y}G\left(X|y\right)=E_{y}G\left(p_{X|y}\right)$$

gives the average minimum number of guesses to find *X* after having observed *Y*. Additionally:

(22) $$R_{2}\left(X|Y\right)≜E_{y}R_{2}\left(X|y\right)=E_{y}R_{2}\left(p_{X|y}\right)$$

gives the average probability of non-collision to identify *X* upon observation of *Y*, and

(23) $$P_{e}\left(X|Y\right)≜E_{y}P_{e}\left(X|y\right)=1-E_{y}\max p_{X|y}$$

gives the minimum average probability of error, as achieved by the maximum a posteriori (MAP) decision rule. The “conditional statistical randomness” is likewise defined as shown:

(24) $$R\left(X|Y\right)≜E_{y}R\left(X|y\right)=E_{y}R\left(p_{X|y}\right).$$

For the generalized quantities of Section 1.5, the conditional ρ-guessing entropy is given by the following:

(25) $$G_{\rho }\left(X|Y\right)≜E_{y}G_{\rho }\left(X|y\right)=E_{y}G_{\rho }\left(p_{X|y}\right)$$

and the conditional *m*th-order probability of error is as below:

(26) $$P_{e}^{m}\left(X|Y\right)≜E_{y}P_{e}^{m}\left(X|y\right)=E_{y}P_{e}^{m}\left(p_{X|y}\right).$$

For α-entropy, however, many different definitions of conditional α-entropy have been proposed in the literature [15]. The preferred choice for most applications seems to be Arimoto’s definition [16]:

(27) $$H_{\alpha }\left(X|Y\right)≜\frac{\alpha }{1-\alpha }\log E_{y}{∥p_{X|y}∥}_{\alpha },$$

where the expectation over *Y* is taken on the α-norm inside the logarithm and not outside. Shannon’s conditional entropy H(X|Y) is recovered in the limiting case α→1. One nice property of Arimoto’s definition is that it is compatible with that of $P_{e}\left(X|Y\right)$ in the limiting case α→∞, since the relation $H_{\infty }=\log\frac{1}{1-P_{e}}$ of (6) naturally extends to conditional quantities:

(28) $$H_{\infty }\left(X|Y\right)=\log\frac{1}{1-P_{e}\left(X|Y\right)}.$$

Notice that for any order α≠1, Arimoto’s definition can be rewritten as a simple expectation of $\varphi_{\alpha }\left(H_{\alpha }\right)$ instead of $H_{\alpha }$:

(29) $$\varphi_{\alpha }\left(H_{\alpha }\left(X|Y\right)\right)=E_{y}\varphi_{\alpha }\left(H_{\alpha }\left(p_{X|y}\right)\right),$$

where $\varphi_{\alpha }$ is the increasing function, defined as follows:

(30) $$\varphi_{\alpha }\left(x\right)≜\mathrm{sgn}\left(1-\alpha \right)\exp\left(\frac{1-\alpha }{\alpha }x\right).$$

The requirement that $\varphi_{\alpha }$ is increasing is important in the following. The signum term was introduced so that $\varphi_{\alpha }$ is increasing, not only for 0<α<1, but also for α>1. The exponential function exp is assumed to the same base as the logarithm: $\exp x=2^{x}$ for *x* in bits, $10^{x}$ in dits, $e^{x}$ in nats). In what follows, we indifferently refer to $H_{\alpha }$ or $\varphi_{\alpha }\left(H_{\alpha }\right)$.

### 1.9. Aim and Outline

The enumeration in the preceding subsections is by no means exhaustive. Every subfield or application has its preferred criterion, either information/estimation theoretic or statistical, conditioned on some observations or not. Clearly, all these randomness measures share many properties.

Therefore, a natural question is to determine a (possibly minimal) set of properties that characterize all possible randomness measures. Many axiomatic approaches have been proposed for entropy [1,17], α-entropy [9], information leakage [18] or conditional entropy [19,20].

Extending the work in [21], Section 2 presents a simple alternative, which naturally encompass all common randomness measures *H*, $H_{\alpha }$, *G*, $G_{\rho }$, $P_{e}$, $P_{e}^{m}$, $R_{2}$ and *R*, based on two natural axioms:

- Equivalent random variables are equally random;
- Knowledge reduces randomness (on average).

Many properties, shared by all randomness measures described above, are deduced from these two axioms.

Another important issue is to study the relationship between randomness measures, by establishing the exact locus or joint range of two such measures among all probability distributions with tight lower and upper bounds. In this paper, extending the presentation made in [21], we establish the optimal bounds relating information-theoretic (e.g., entropic) quantities on one hand and statistical quantities (probability of error and statistical distance) on the other hand.

Section 3 establishes general optimal Fano and reverse-Fano inequalities, relating any randomness measure to the probability of error. This generalizes Fano’s original inequality [22] $H\left(X|Y\right)\leq \left(1\;-\;P_{e}\left(X|Y\right)\right)\log\frac{1}{1\;-\;P_{e}\left(X|Y\right)}+P_{e}\left(X|Y\right)\log\frac{M-1}{P_{e}\left(X|Y\right)}$, which has become ubiquitous in information theory (e.g., to derive converse channel coding theorems) and in statistics (e.g., to derive lower bounds on the maximum probability of error in multiple hypothesis testing).

Section 4 establishes general optimal Pinsker and reverse-Pinsker inequalities, relating any randomness measure to the statistical randomness or the statistical distance to the uniform. Generally speaking, Pinsker and reverse-Pinsker inequalities relate some divergence measure (e.g., d(p∥q) or $d_{\alpha }\left(p∥q\right)$) between two distributions to their statistical distance Δ(p,q). Here, following the discussion in Section 1.6, we restrict ourselves to the divergence or distance to the uniform distribution q=u. (For the general case of arbitrary distributions p,q see, e.g., the historical perspective on Pinsker–Schützenberger inequalities in [23].). In this context, we improve the well-known Pinsker inequality [24,25], which reads $D\left(p∥u\right)=\log M-H\left(p\right)\geq {2\log e\cdot ∥p-u∥}_{1}^{2}$. This inequality, of more general applicability for any distributions p,q, is no longer optimal in the particular case q=u.

Finally, Section 5 lists some applications in the literature, and Section 6 gives some research perspectives.

## 2. An Axiomatic Approach

Let *X* be any *M*-ary random variable with distribution $p_{X}$. How should a measure of “randomness” R(X)∈R of *X* be defined in general? To simplify the discussion, we assume that R(X)≥0 is nonnegative.

As advocated by Shannon [26], such a notion should not depend on the particular “reversible encoding” of *X*. In other words, any two equivalent random variables should have the same measure R(X), where equivalence is defined as follows.

**Definition 1** (Equivalent Variables)**.** *Two random variables X and Y are* equivalent: X≡Y, *if there exist two mappings f and g, such that Y=f(X) a.s. (almost surely, i.e., with probability one) and X=g(Y) a.s.*

**Remark 1** (Equivalent Measures)**.** *Obviously, it is also essentially equivalent to study R(X) or $R{\left(X\right)}^{2}$, for example, or any quantity of the form φ(R(X)), where $\varphi :R_{+}\to R_{+}$ is any increasing (invertible) function.*

**Definition 2** (Conditional Randomness)**.** *Given any random variable Y, the conditional form of R is defined as follows:*

(31) $$R\left(X|Y\right)=E_{y}R\left(X|y\right)$$

*where X|y (or X|Y=y) denotes the random variable X, conditioned of the event Y=y. This quantity represents the average amount of randomness of X knowing Y.*

**Remark 2** (Equivalent Conditional Measures)**.** *Again, it is essentially equivalent to study R(X|Y) or φ(R(X|Y)), where $\varphi :R_{+}\to R_{+}$ is any increasing function. One may, therefore, generalize the notion of conditional randomness by writing $\varphi \left(R\left(X|Y\right)\right)=E_{y}\varphi \left(R\left(X|y\right)\right)$ in place of (31), the same as (29) for α-entropy. However, in the sequel, we stay with the basic Definition 2 and simply assume that φ(R) is considered instead of R whenever it is convenient to do so.*

In the sequel, we study the implications of only two axioms:

**Axiom 1** (Equivalence)**.** *
X≡Y⇒R(X)=R(Y)
*

**Axiom 2** (Knowledge Reduces Randomness)**.**
(32) R(X|Y)≤R(X).

We find such postulates quite intuitive and natural. First, equivalent random variables should be equally random. Second, knowledge of some side observation should, *on average*, reduces randomness.

All randomness quantities described in Section 1 obviously satisfy Axiom 1. That they also satisfy Axiom 2 is shown in the following examples.

**Example 1** (Entropies)**.** *For Shannon’s entropy H, the inequality H(X|Y)≤H(X) is well known Thm 2.6.5 in [2]. This is often paraphrased as “conditioning reduces entropy”, “knowledge reduces uncertainty” or “information can’t hurt”. The difference H(X)−H(X|Y)=I(X;Y) is the mutual information, which is always nonnegative. Inequality $H_{\alpha }\left(X|Y\right)\leq H_{\alpha }\left(X\right)$ is also known to hold for any α>0, see [15,16] and Example 4 below.*

**Example 2** (Guessing Entropies)**.** *Axiom 2 for the guessing entropies G or $G_{\rho }$ can be easily checked from their definition, as follows.*

*Let N∈N={1,2,…} be any random variable giving the number of guesses needed to find X in any guessing strategy. N is equivalent to X (Definition 1) since every value of N corresponds to a unique value of X, and vice versa. By definition, $G_{\rho }\left(X\right)=\min_{N\equiv X}E\left(N^{\rho }\right)$, where the minimum is over all possible N∈N equivalent to X (corresponding to all possible strategies). Now, $G_{\rho }\left(X|Y\right)=E_{y}G_{\rho }\left(X|y\right)\leq E_{y}E\left(N^{\rho }|y\right)=E\left(N^{\rho }\right)$, by the law of total expectation. Taking the minimum over N≡X gives $G_{\rho }\left(X|Y\right)\leq G_{\rho }\left(X\right)$, which is Axiom 2.*

*The case ρ=1 was already shown in [27]. The result is quite intuitive: any side information Y can only improve the guess of X.*

**Example 3** (Error Probabilities)**.** *Axiom 2 for the error probability $P_{e}=P_{e}^{1}$ follows from the corresponding inequality for $H_{\infty }=\log\frac{1}{1-P_{e}}$ (see (28) and Example 1 for α=∞), but it can also be checked directly from its definition, as well as in the case of $P_{e}^{m}$ of order m, as follows.*

*The mth order error probability is $P_{e}^{m}\left(X\right)=\min_{{\hat{x}}_{1},\ldots ,{\hat{x}}_{m}}P\left(X\neq {\hat{x}}_{1},X\neq {\hat{x}}_{2},\ldots ,X\neq {\hat{x}}_{m}\right)$, i.e., the minimum probability that X is not equal to any of the m first estimates ${\hat{x}}_{1},{\hat{x}}_{2},\ldots ,{\hat{x}}_{m}$. Then, $P_{e}^{m}\left(X|Y\right)=E_{y}\min_{{\hat{x}}_{1},\ldots ,{\hat{x}}_{m}}P\left(X\neq {\hat{x}}_{1},\ldots ,X\neq {\hat{x}}_{m}|y\right)\leq E_{y}P\left(X\neq {\hat{x}}_{1},\ldots ,X\neq {\hat{x}}_{m}|y\right)=P\left(X\neq {\hat{x}}_{1},\ldots ,X\neq {\hat{x}}_{m}\right)$, by the law of total probability, for every sequence ${\hat{x}}_{1},\ldots ,{\hat{x}}_{m}$. Taking the minimum over such sequences gives $P_{e}^{m}\left(X|Y\right)\leq P_{e}^{m}\left(X\right)$, which is Axiom 2.*

*The case m=1 was already shown, e.g., in [27]. Again, the result is quite intuitive: any side information Y can only improve the estimation of X.*

### 2.1. Symmetry and Concavity

We now rewrite Axioms 1 and 2 as equivalent conditions on probability distributions.

**Definition 3** (Probability “Simplex”)**.** *Let P be the set of all sequences of nonnegative numbers:*

(33) $$p=(p_{1},p_{2},p_{3},\ldots )$$

*such that the following are satisfied:*

- *Only a finite number of them are positive: $p_{k}\neq 0$ for finitely many k;*
- *They sum to 1: $\sum_{k}p_{k}=1$.*

Notice that P has infinite dimension even though only a finite number of components are nonzero in every p∈P. Thus, any p∈P can be seen as the probability distribution of M-ary random variables with arbitrary large M.

**Theorem 1** (Symmetry)**.** *Axiom 1 is equivalent to the condition that R(X)=R(p) is a symmetric function of $p=(p_{1},p_{2},p_{3},\ldots )\in P$, identified as the probability distribution of X.*

**Proof.**  Let X be the finite set (“alphabet”) of all values taken by $X∼p_{X}$, and let f be an injective mapping from X to N={1,2,…}, whose image is a finite subset of N. From Definition 1, X is equivalent to f(X)∈N, with probabilities $p=(p_{1},p_{2},\ldots )$. Then, by Axiom 1, R(X) does not depend on the particular values of X but only on the corresponding probabilities, so that R(X)=R(p), where p∈P is identified to $p_{X}$. Now, letting h be any bijection (permutation) of N, Axiom 1 implies that R(p) does not depend on the ordering of the $p_{k}$s, that is, R(p) is a symmetric function of p. Conversely, any bijection applied to X can only change the ordering of the $p_{k}$s in $p=p_{X}$, which leaves R(p)=R(X) as invariant. □

Accordingly, it is easily checked directly that all expressions in terms of probability distributions p of random measures given in Section 1 are symmetric in p.

**Remark 3.**  *Some authors [17] define P as the union of all $P_{M}$ for M∈N, where $P_{M}$ is the M-simplex $\left\{\left(p_{1},p_{2},\ldots ,p_{M}\right),p_{k}\geq 0,p_{1}+\cdots +p_{M}=1\right\}$. With this viewpoint, even when the expression of R(p) does not explicitly depend on M, one has to define R(p) separately for all different values of M as a function $R_{M}\left(p_{1},p_{2},\ldots ,p_{M}\right)$, defined over $P_{M}$, and further impose the compatibility condition that $R_{M+1}\left(p_{1},p_{2},\ldots ,p_{M},0\right)=R_{M}\left(p_{1},p_{2},\ldots ,p_{M}\right)$, as in [17] (this is called “expansibility” in [20]).*

*Such expansibility condition is unnecessary to state explicitly in our approach: it is an obvious consequence of an appropriate choice of f in Definition 1, namely, the injective embedding of {1,2,…,M} into {1,2,…,M+1}.*

**Theorem 2** (Concavity)**.** *Axiom 2 is equivalent to the condition that R(p) is concave in p.*

**Proof.**  Using the notations of Theorem 1, Definition 2 and (19), Axiom 2 can be rewritten as shown:

(34) $$E_{y}R\left(p_{X|y}\right)\leq R\left(p_{X}\right)=R\left(E_{y}p_{X|y}\right).$$

This is exactly Jensen’s inequality for concave functions on the convex “simplex” P. □

**Remark 4**  (φ-Concavity)**.** *Similarly as in Remark 2, we may consider φ(R) in place of R in the definition of conditional randomness, where $\varphi :R_{+}\to R$ is any increasing function. Then, by Theorem 2, φ(R) is concave, that is, R(p) is a φ-concave function of p (for example, for φ=log, one recovers the usual definition of a log-concave function). This is called “core-concavity” in [20].*

**Example 4** (Symmetric Concave Measures)**.** *All randomness measures of Examples 1–3 satisfy both Axioms 1 and 2, and are, therefore, symmetric concave in p. This can also be checked directly from certain closed-form expressions given in Section 1:*

- *Shannon’s entropy H, as well as the complementary index of coincidence $R_{2}$, can be written in the form $\sum_{k}r\left(p_{k}\right)$, where r is a strictly concave function. Thus, both are symmetric and strictly concave in p;*
- *Statistical randomness R(p) can also be written in this form, where $r\left(p_{k}\right)=-\frac{1}{2}\left|p_{k}-\frac{1}{M}\right|$ is concave in $p_{k}$. Thus, R(p) is also symmetric concave and, therefore, is also an acceptable randomness measure satisfying Axioms 1 and 2;*
- *For α-entropy, consider $\varphi_{\alpha }\left(H_{\alpha }\left(p\right)\right)=\mathrm{sgn}\left(1-\alpha \right){∥p∥}_{\alpha }$ where $\varphi_{\alpha }$ is the increasing function (30). It is known that the α-norm ${∥\cdot ∥}_{\alpha }$ is strictly convex for finite α>1 (by Minkowski’s inequality) and strictly concave for 0<α<1 (by the reverse Minkowski inequality). Thus, α-entropy is symmetric and (strictly) $\varphi_{\alpha }$-concave in the sense of Remark 4. Therefore, one finds anew that it satisfies Axioms 1 and 2.*

**Corollary 1** (Mixing Increases Randomness)**.** *Let p,q∈P be any two probability distributions and consider the “mixed” distribution $\lambda p+\bar{\lambda }q$, where λ≥0, $\bar{\lambda }\geq 0$, and $\lambda +\bar{\lambda }=1$. Then:*

(35) $$R\left(\lambda p+\bar{\lambda }q\right)\geq \lambda R\left(p\right)+\bar{\lambda }R\left(q\right).$$

*In particular, mixing two equally random distributions R(p)=R(q) results in a “more random” distribution: $R\left(\lambda p+\bar{\lambda }q\right)\geq R\left(p\right)=R\left(q\right)$.*

**Proof.**  Immediate from the concavity of R. □

**Example 5.**  *The mixing property of the Shannon entropy H is well-known Thm. 2.7.3 in [2]. A well-known thermodynamic interpretation is that mixing two gases of equal entropy results in a gas with higher entropy.*

### 2.2. Basic Properties in Terms of Random Variables

In terms of random variables, one can deduce the following properties.

**Corollary 2** (Consistency)**.** *If X is independent of Y, then R(X|Y)=R(X). In particular, let 0 denote any deterministic variable (by Defintion 1, any deterministic random variable is equivalent to the constant 0). Then:*

(36) R(X|0)=R(X).

Thus “absolute” (unconditional) randomness R(X) can be recovered as a special case of conditional randomness.

**Proof.**  If X and Y are independent, then $p_{X|y}=p_{X}$ for (almost) any y, so that $R\left(X|Y\right)=E_{y}R\left(X|y\right)=E_{y}R\left(X\right)=R\left(X\right)$. In particular, X and 0 are always independent. □

**Remark 5** (Strict Concavity)**.** *A randomness measure R is “strictly concave” in p if Jensen’s inequality (34) holds with equality only when $p_{X|y}=p_{X}$ for almost all y. This can be stated in terms of random variables as follows. For any strictly concave random measure R, (32) is strict unless independence holds:*

(37) R(X|Y)=R(X)⇔XisindependentofY.

**Example 6** (Strictly Concave Measures)**.** *As already seen in Example 4, entropy H, all α-entropies $\varphi_{\alpha }\left(H_{\alpha }\right)$ for finite α>0 and $R_{2}$ are strictly concave.*

*In particular, for entropy, H(X|Y)=H(X) if and only if X and Y are independent. This is well known since the mutual information I(X;Y)=H(X)−H(X|Y) vanishes only in the case of independence [2] (p. 28). More generally, for α-entropy, $H_{\alpha }\left(X|Y\right)=H_{\alpha }\left(X\right)$ if and only if X and Y are independent.*

*Guessing entropy G, or, more generally, ρ-guessing entropy $G_{\rho }$, is* not *strictly concave in p. For example, $G_{\rho }\left(1-\varepsilon ,\varepsilon ,0,0,\ldots \right)=1-\varepsilon +2^{\rho }\varepsilon$ is linear in $\varepsilon <\frac{1}{2}$.*

**Corollary 3** (Additional Knowledge Reduces Randomness)**.** *Inequality (32) is equivalent to the following:*

(38) R(X|Y,Z)≤R(X|Y)

*for any Y,Z.*

**Proof.**  Inequality (32) applied to X|y and Z for fixed y gives $R\left(X|y,Z\right)=E_{z|y}R\left(p_{X|y,z}\right)\leq R\left(p_{X|y}\right)=R\left(X|y\right)$. Taking the expectation over Y of both sides yields the announced inequality. Conversely, letting Y=0, one obtains R(X|Z)≤R(X), which is (32). □

**Corollary 4** (Data Processing Inequality: Processing Knowledge Increases Randomness)**.** *For any Markov chain X−Y−Z (i.e., such that $p_{X|Y,Z}=p_{X|Y}$), one has the following:*

(39) R(X|Y)≤R(X|Z).

*This property is equivalent to (32).*

**Proof.**  Since $p_{X|Y}=p_{X|Y,z}$ for (almost) any z, one has R(X|Y)=R(X|Y,z)=R(X|Y,Z), which, from Corollary 3, is ≤R(X|Z). Conversely, letting Z=0, one recovers (32). □

**Example 7** (Data Processing Inequalities)**.** *For entropy H, the property H(X|Y)≤H(X|Z) amounts to I(X;Z)≤I(X;Y), i.e., (post-)processing in the Markov chain X−Y−Z can never increase information § 2.8 in [2]. The data processing inequality for $P_{e}$ and G was already shown in [27].*

### 2.3. Equalization (Minorization) via Robin Hood Operations

We now turn to another type of “mixing” probability distributions which are sometimes known as Robin Hood operations. To quote Arnold [28]:

“When Robin and his merry hoods performed an operation in the woods they took from the rich and gave to the poor. The Robin Hood principle asserts that this decreases inequality (subject only to the obvious constraint that you don’t take too much from the rich and turn them into poor.)”

**Definition** **4** (Robin Hood operations [28])**.** *An* elementary *“Robin Hood” operation p↦q in P modifies only two probabilities $\left(p_{i},p_{j}\right)\mapsto \left(q_{i},q_{j}\right)$ (i≠j) in such a way that $|p_{i}-p_{j}\left|\geq \right|q_{i}-q_{j}|$. A (general) “Robin Hood operation” results from a finite sequence of elementary Robin Hood operations.*

Notice that in an elementary Robin Hood operation, the sum $p_{i}+p_{j}=q_{i}+q_{j}$ should remain the same, since p and q are probability distributions. The fact that $|p_{i}-p_{j}|$ decreases “increases equality”, i.e., makes the probabilities more equal. This can be written as follows:

(40) $$\left\{\begin{array}{c} q_{i}=p_{i}-\delta \\ q_{j}=p_{j}+\delta \end{array}\right.$$

*provided that $\left|\delta |\leq \right|p_{i}-p_{j}|$ (“you don’t take too much from the rich and turn them into poor”). Setting $\lambda =1-\frac{\delta }{p_{i}-p_{j}}\in \left[0,1\right]$, (40) can be easily rewritten in the form:*

(41) $$\left\{\begin{array}{c} q_{i}=\lambda p_{i}+\bar{\lambda }p_{j}\\ q_{j}=\bar{\lambda }p_{i}+\lambda p_{j} \end{array}\right.$$

where λ≥0, $\bar{\lambda }\geq 0$ and $\lambda +\bar{\lambda }=1$.

**Remark 6** (Increasing Probability Product)**.** *In any elementary Robin Hood operation $\left(p_{i},p_{j}\right)\mapsto \left(\lambda p_{i}+\bar{\lambda }p_{j},\bar{\lambda }p_{i}+\lambda p_{j}\right)$, the product:*

(42) $$q_{i}q_{j}=\left(\lambda p_{i}+\bar{\lambda }p_{j}\right)\left(\bar{\lambda }p_{i}+\lambda p_{j}\right)=p_{i}p_{j}+\lambda \bar{\lambda }{\left(p_{i}-p_{j}\right)}^{2}\geq p_{i}p_{j}$$

*always increases, with equality if and only if either λ=0 or 1, or else $p_{i}=p_{j}$. This equality condition boils down to $|p_{i}-p_{j}\left|=\right|q_{i}-q_{j}|$, that is, the unordered set $\left\{p_{i},p_{j}\right\}=\left\{q_{i},q_{j}\right\}$ is unchanged.*

*Therefore, in any general Robin Hood operation, the product of all modified probabilities always increases, unless the probability distribution is unchanged (up to the order of the probabilities).*

**Remark 7** (Inverse Robin Hood Operation)**.** *One can also define a “Sheriff of Nottingham” operation as an inverse Robin Hood operation, resulting from a finite sequence of elementary Sheriff of Nottingham operations of the form $\left(p_{i},p_{j}\right)\mapsto \left(q_{i},q_{j}\right)$, where $|p_{i}-p_{j}\left|\leq \right|q_{i}-q_{j}|$. Increasing the quantity $|p_{i}-p_{j}|$ “increases inequality”, i.e., makes the probabilities more unequal.*

**Definition 5** (Equalization Relation)**.** *We write X⪯Y (“X is equalized by Y”) if $p_{Y}$ can be obtained from $p_{X}$ by a Robin Hood operation. Such operation “equalizes” $p_{X}$ in the sense that $p_{Y}$ is “more equal” or “more uniform” than $p_{X}$. In terms of distributions, we also write $p_{X}⪯p_{Y}$. Equivalently, $p_{X}$ can be obtained from $p_{Y}$ by a Sheriff of Nottingham operation ($p_{X}$ is more unequal than $p_{Y}$). We may also write Y⪰X or $p_{Y}⪰p_{X}$.*

**Remark 8** (Generalization)**.** *The above definitions hold verbatim for any vector or finitely many nonnegative numbers $p_{k}$ with a fixed sum $s=\sum_{k}p_{k}$ (not necessarily equal to one). In the following, we sometimes use the concept of “equalization” in this slightly more general context.*

**Remark 9** (Minorization)**.** *X⪯Y amounts to saying that $p_{X}$ “majorizes” $p_{Y}$ in majorization theory [28,29]. So, in fact, the equalization relation ⪯ is a “minorization”—the opposite of a majorization. Unfortunately, it is common in majorization theory to write “Y⪯X” when X “majorizes” Y, instead of X⪯Y when Y is “more equal” than X. Arguably, the notation adopted in this paper is more convenient, since it follows the usual relation order between randomness measures such as entropy.*

*Also notice that the present approach avoids the use of Lorenz order [28,29] and focuses on the more intuitive Robin Hood operations.*

**Remark 10** (Partial Order)**.** *It is easily seen that ⪯ is a partial order on the set of (finitely valued) discrete random variables (considering two variables “equal” if they are equivalent in the sense of Definition 1). Indeed, reflexivity and transitivity are immediate from the definition, and antisymmetry is, e.g., an easy consequence of Remark 6: if X⪯Y and Y⪯X, then the product of all modified probabilities of X cannot increase by the two combined Robin Hood operations. Therefore, $p_{Y}$ should be the same as $p_{X}$ up to order; hence, X≡Y.*

The following fundamental lemmas establish expressions for maximally equal and unequal distributions.

**Lemma 1** (Maximally Equal = Uniform)**.** *For any vector $p=(p_{1},p_{2},\ldots ,p_{M})$ of nonnegative numbers with sum $s=\sum_{k}p_{k}$:*

(43) $$p⪯(\frac{s}{M},\frac{s}{M},\ldots ,\frac{s}{M}).$$

*In particular, any probability distribution p is equalized by the uniform distribution u:*

(44) p⪯u

**Proof.**  Suppose at least one component of *p* is $\neq \frac{s}{M}$. Since the $p_{k}$s sum to *s*, there should be at least one $p_{i}>\frac{s}{M}$ and one $p_{j}<\frac{s}{M}$. By a suitable Robin Hood operation on $\left(p_{i},p_{j}\right)$, at least one of these two probabilities can be made $=\frac{s}{M}$, reducing the total number of components $\neq \frac{s}{M}$. Continuing in this manner, we arrive at all probabilities equal to $\frac{s}{M}$ after, at most, M−1 Robin Hood operations. □

**Lemma 2** (Maximally Unequal)**.** *For any vector $p=(p_{1},p_{2},\ldots ,p_{M})$ of nonnegative numbers with sum $s=\sum_{k}p_{k}$ and constrained maximum $\max_{k}p_{k}\leq P$:*

(45) p⪰(P,…,P,r,0,…,0)

*with remainder component $r=s-\lfloor \frac{s}{P}\rfloor P$. Without the maximum constraint (P=s), one simply has the following:*

(46) p⪰(s,0,…,0).

*In particular, for any probability distribution p:*

(47) p⪰δ

*where δ is the (Dirac) probability distribution of any deterministic variable. (This can be written in terms of random variables as X⪰0, since, by Defintion 1, any deterministic random variable is equivalent to the constant 0.)*

**Proof.**  Suppose at least two components lie between 0 and *P*: $0<p_{i},p_{j}<P$. By a suitable Sheriff of Nottingham operation on $\left(p_{i},p_{j}\right)$, at least one of these two probabilities can be made either =0 or =P, reducing the number of components lying inside (0,P). Continuing in this manner, we arrive at, at most, one component r∈(0,P). Finally, the sum constraint implies s=qP+r where 0<r<P, whence $q=\lfloor \frac{s}{P}\rfloor$. □

**Theorem 3**  (Schur Concavity [28,29])**.**

(48) X⪯Y⇒R(X)≤R(Y)

**Proof.**  It suffices to prove the inequality for an elementary Robin Hood operation $\left(p_{i},p_{j}\right)\mapsto \left(\lambda p_{i}+\bar{\lambda }p_{j},\bar{\lambda }p_{i}+\lambda p_{j}\right)$. Dropping the dependence on the other (fixed) probabilities, one has, by symmetry, (Theorem 1) and concavity (Theorem 2):

(49) $$R\left(p_{i},p_{j}\right)=\lambda R\left(p_{i},p_{j}\right)+\bar{\lambda }R\left(p_{j},p_{i}\right)\leq R\left(\lambda p_{i}+\bar{\lambda }p_{j},\lambda p_{j}+\bar{\lambda }p_{i}\right).$$

□

Inequality (48), expressed in terms of distributions:

(50) $$p_{X}⪯p_{Y}\Rightarrow R\left(p_{X}\right)\leq R\left(p_{Y}\right)$$

is known as “Schur concavity” [28,29].

**Remark 11.**  *Theorem 3 can also be given a physical interpretation similar to Corollary 1. In fact, from (41), any Robin Hood operation can be seen as mixing two permuted probability distributions, which have equal randomness. Such mixing can only increase randomness.*

**Example 8** (Entropy is Schur-Concave)**.** *That the Shannon entropy is Schur-concave is well known § 13 E in [29]. Similar to concavity (Example 5), this also has a similar physical interpretation: a liquid mixed with another results in a “more disordered”, “more chaotic” system, which results in a “more equal” distribution and a higher entropy § 1 A9 in [29].*

**Remark 12**  (φ-Schur Concavity)**.** *Schur concavity is not equivalent to concavity (even when assuming symmetry). In fact, with the notations of Remark 4, it is obvious that Schur concavity of R is equivalent to Schur concavity of φ(R), where $\varphi :R_{+}\to R_{+}$ is any increasing function. In other words, while “φ-concavity” (in the sense of Remark 4) is not the same as concavity, there is no need to introduce “φ-Schur concavity”, since it is always equivalent to Schur concavity.*

**Remark 13** (Strict Schur Concavity)**.** *A randomness measure R is “strictly Schur concave” if the inequality R(X)≤R(Y) for X⪯Y holds with equality R(X)=R(Y) if and only if X≡Y.*

*If R(p) is strictly concave (see Remark 5), then equality holds in (49) if and only if either λ=0 or 1, or else $p_{i}=p_{j}$. Either of these conditions means that $\left\{p_{i},p_{j}\right\}$ is unchanged. Therefore, in this case, R is also strictly Schur concave.*

*Remark 6 states that the product of nonzero probabilities is strictly Schur-concave.*

**Example 9** (Strictly Schur Concave Measures)**.** *Randomness measures presented in Section 1 are (Schur) concave, but not all of them are* strictly *Schur concave:*

- *Not only the Shannon entropy H is Schur concave (Example 8), but, as seen in Example 6, H, as well as all α-entropies $\varphi_{\alpha }\left(H_{\alpha }\right)$ for finite α>0 and $R_{2}$, are strictly concave and, hence, strictly Schur concave;*
- *As seen also in Example 6, guessing entropy G, or, more generally, ρ-guessing entropy $G_{\rho }$, is* not *strictly concave in p. However, G and $G_{\rho }$ are strictly Schur concave by the following argument.*
  *It suffices to show that some elementary Robin Hood operation (40) $\left(p_{i},p_{j}\right)\mapsto \left(p_{i}-\delta ,p_{j}+\delta \right)$ (with δ≠0) strictly increases $G_{\rho }$. One may always choose δ as small as one pleases, since any elementary Robin Hood operation on $\left(p_{i},p_{j}\right)$ can be seen as resulting from other ones on $\left(p_{i},p_{j}\right)$ with smaller δ. One chooses δ small enough such that the elementary Robin Hood operation does not change the order of the probabilities in p. With the notations of Section 1.2, assuming, for example, that $p_{i}=p_{\left(i\right)}>p_{j}=p_{\left(j\right)}$, where i<j, then δ>0 and $i^{\rho }p_{\left(i\right)}+j^{\rho }p_{\left(j\right)}<i^{\rho }\left(p_{\left(i\right)}-\delta \right)+j^{\rho }\left(p_{\left(j\right)}+\delta \right)$, since $j^{\rho }>i^{\rho }$. This shows that $G_{\rho }$ strictly increases;*
- *Error probability $P_{e}$, or, more generally, $P_{e}^{m}$, is neither strictly concave nor strictly Schur concave in general. In fact, if M≥m+2, any elementary Robin Hood operation on $p_{i},p_{j}<p_{\left(m\right)}$ leaves $P_{e}^{m}$ unchanged;*
- *Statistical randomness R is neither strictly concave nor strictly Schur concave if M>2. For example, it is easily checked from the definition (18) that the elementary Robin Hood operation $\left(\frac{1}{M},\frac{2}{M}\right)\mapsto \left(\frac{4/3}{M},\frac{5/3}{M}\right)$ leaves R unchanged.*

### 2.4. Resulting Properties in Terms of Random Variables

**Corollary 5** (Minimal and Maximal Randomness)**.**
(51) R(δ)≤R(X)≤R(u)

*In other words, minimal randomness is achieved for X=0 (for any deterministic variable 0) and maximal randomness is achieved for uniformly distributed X.*

**Proof.**  From Lemmas 1 and 2, one obtains $\delta ⪯p_{X}⪯u$. The result follows by Theorem 3. □

**Remark 14** (Zero Randomness)**.** *Without loss of generality, we may always impose that R(0)=0 by considering R(X)−R(0) in place of R(X). Then, zero randomness is achieved when X≡0. It is easily checked from the expressions given in Section 1 that this convention holds for H, $H_{\alpha }$, logG, $\log G_{\rho }$, $P_{e}$, $P_{e}^{m}$, $R_{2}$ and R.*

*To simplify notations in the remainder of this paper, we assume that the zero randomness convention R(0)=0 always holds.*

**Example 10** (Distribution Achieving Zero Randomness)**.** *By Remark 13, if R is strictly Schur concave, zero randomness is achieved only when X≡0:*

(52) R(X)=0⇔X≡0.

- *As seen in Example 9, this is the case for H, $H_{\alpha }$, logG, $\log G_{\rho }$ and $R_{2}$. In particular, we recover the well known property that zero entropy is achieved only when X is deterministic;*
- *Although the error probability is not strictly Schur concave, one can check directly that $P_{e}\left(p\right)=0$ if and only if $p_{\left(1\right)}=1$, which corresponds to the δ distribution;*
- *Similarly, from the discussion in Section 1.7, R(p)=0 correspond to the maximum value of $\Delta \left(p,u\right)=1-\frac{1}{M}$ attained for $K=|T_{+}|=1$ and $P(T_{+})=1$, which, again, corresponds to a δ distribution.*

*To summarize, all quantities H, $H_{\alpha }$, logG, $\log G_{\rho }$, $P_{e}$, $R_{2}$ and R satisfy (52).*

**Remark 15** (Maximal Randomness Increases with *M*)**.** *For an M-ary random variable, maximal randomness $R_{M}=R\left(u_{M}\right)$ is attained for a uniform distribution $u_{M}=\left(\frac{1}{M},\frac{1}{M},\ldots ,\frac{1}{M}\right)$. Since, by Lemma 1, $u_{M}⪯u_{M+1}$, one has $R_{M}\leq R_{M+1}$: maximal randomness $R_{M}$ increases with M.*

**Example 11** (Distribution Achieving Maximum Randomness)**.** *The following maximum values for M-ary random variables are easily checked from the expressions given in Section 1:*

- *maxH=H(u)=logM, and, more generally, $\max H_{\alpha }=H_{\alpha }\left(u\right)=\log M$. Since H and $H_{\alpha }$ are strictly Schur-concave, the maximum $H_{\alpha }\left(X\right)=\log M$ is attained if and only if X is uniformly distributed. This observation is also an easy consequence of (10) or (11);*
- *$\max G=G\left(u\right)=\frac{M+1}{2}$, $\max G_{2}=G_{2}\left(u\right)=\frac{\left(M+1/2)(M+1\right)}{3}$, $\max G_{3}=G_{3}\left(u\right)=\frac{M{\left(M+1\right)}^{2}}{4}$, etc. Again, since G and $G_{\rho }$ are strictly Schur-concave, their maximum is achieved if and only if X is uniformly distributed;*
- *$\max P_{e}=P_{e}\left(u\right)=1-\frac{1}{M}$, and, more generally, $\max P_{e}^{m}=P_{e}^{m}\left(u\right)=1-\frac{m}{M}$. The maximum of $P_{e}\left(X\right)$ is achieved if and only if the maximum probability $p_{\left(1\right)}$ equals $\frac{1}{M}$, which implies that X is uniformly distributed;*
- *$\max R_{2}=\max R=1-\frac{1}{M}$ (see (12) and (18)) is achieved if and only if p=u.*

*To summarize, for all quantities H, $H_{\alpha }$, logG, $\log G_{\rho }$, $P_{e}$, $R_{2}$ and R, the unique maximizing distribution is the uniform distribution. Notice that, as expected, each of these maximum values increases with M.*

**Corollary 6** (Deterministic Data Processing Inequality: Processing Reduces Randomness)**.** *For any deterministic function f:*

(53) R(f(X))≤R(X).

**Proof.**  Consider preimages by f of values y=f(x). The application of f can be seen as resulting from a sequence of elementary operations, each of which puts together two distinct values of x (say, $x_{i}$ and $x_{j}$) in the same preimage of some y. In terms of probability distributions, this amounts to a Sheriff of Nottingham operation $\left(p_{i},p_{j}\right)\mapsto \left(p_{i}+p_{j},0\right)$. Overall, one has f(X)⪯X. The result then follows by Schur concavity (Theorem 3). □

**Example** **12.** *The fact that H(f(X))≤H(X) is well known (see Ex. 2.4 in [2]). This can also be seen from the data processing inequality of Corollary 4 by noting that, since X−f(X)−f(X) is trivially a Markov chain, H(f(X))=I(f(X);f(X))≤I(X;f(X))≤H(X).*

**Remark 16** (Lattices of Information and Majorization)**.** *Shannon [26] defined the order relation X≤Y if X=g(Y) a.s. and showed that it satisfies the properties of a lattice, called the “ìnformation lattice” (see [30] for detailed proofs). With this notation, (53) writes as shown:*

(54) X≤Y⇒R(X)≤R(Y).

*Majorization (or the order relation X⪯Y) also satisfies the properties of a lattice—the “majorization lattice”, as studied in [31]. From the proof of Corollary 6, one actually obtains the following:*

(55) X≤Y⇒X⪯Y⇒R(X)≤R(Y).

*Therefore, the majorization lattice is denser than the information lattice.*

**Corollary 7** (Addition Increases Randomness)**.**
(56) R(X)⪯R(X,Y)

*This property is equivalent to (53).*

**Proof.**  Apply Corollary 6 to the projection f(x,y)=x. Conversely, (53) follows from (56), by taking Y=f(X) and noting that (X,f(X))≡X. □

**Corollary 8** (Total Dependence)**.** *Assuming the zero randomness convention (Remark 14), if (52) holds, then the following holds:*

(57) R(X|Y)=0⇔X=f(Y)a.s.,

*that is, R(X|Y)=0⇔X≤Y in the sense of Shannon (Remark 16).*

**Proof.**  Since R(X|y)≥0 for any y, $R\left(X|Y\right)=E_{y}R\left(X|y\right)=0$ if and only if R(X|y)=0 for (almost) all y. By (52), this implies that X is deterministic given Y=y, i.e., X is a deterministic function of Y. □

**Example** **13.** *From Example 10, (57) is true for H, $H_{\alpha }$, logG, $\log G_{\rho }$, $P_{e}$, $R_{2}$ and R.*

- *The equivalence H(X|Y)=0⇔X=f(Y)a.s. is well known ([2], Ex. 2.5). Knowledge of Y removes equivocation only when X is fully determined by Y;*
- *logG(X|Y)=0⇔G(X|Y)=1⇔X=f(Y)a.s. is intuitively clear: knowing Y allows one to fully determine X in only one guess;*
- *$P_{e}\left(X|Y\right)=0\Leftrightarrow X=f\left(Y\right)\;a.s.$: knowing Y allows one to estimate X without error only when X is fully determined by Y.*

## 3. Fano and Reverse-Fano Inequalities

**Definition 6** (Fano-type inequalities)**.** *A “Fano inequality” (resp. “reverse Fano inequality”) for R(X) gives an upper (resp. lower) bound of R(X) as a function of the probability of error $P_{e}\left(X\right)$. Fano and reverse-Fano inequalities are similarly defined for conditional randomness R(X|Y), lower or upper bounded as a function of $P_{e}\left(X|Y\right)$.*

In this section, we establish optimal Fano and reverse-Fano inequalities, where upper and lower bounds are tight. In other words, we determine the maximum and minimum of R for fixed $P_{e}$. The exact locus of the region $p\in P_{M}\mapsto \left(P_{e}\left(p\right),R\left(p\right)\right)=\left(P_{e}\left(X\right),R\left(X\right)\right)$, as well as the exact locus of all attainable values of $\left(P_{e}\left(X|Y\right),R\left(X|Y\right)\right)$, is determined analytically for fixed M, based on the following.

**Lemma** **3.** *Let $P_{e}=P_{e}\left(p\right)$ and $P_{s}=1-P_{e}$. For any M-ary probability distribution $p\in P_{M}$:*

(58) $$\left(\underset{\left\lfloor \frac{1}{P_{s}}\right\rfloor \;times}{\underbrace{P_{s},\ldots ,P_{s}}},1\;-\;\left\lfloor \frac{1}{P_{s}}\right\rfloor P_{s},0,\ldots ,0\right)⪯p⪯\left(P_{s},\frac{P_{e}}{M-1},\ldots ,\frac{P_{e}}{M-1}\right).$$

**Proof.**  On the left side, apply Lemma 2 with $P=\max p=p_{\left(1\right)}=P_{s}$ and s=1. On the right side, with $p_{\left(1\right)}=P_{s}$ being fixed, apply Lemma 1 to the M−1 remaining probabilities $\left(p_{\left(2\right)},\ldots ,p_{\left(M\right)}\right)$, which sum to $s=1-P_{s}=P_{e}$. □

**Theorem** **4** (Optimal Fano and Reverse-Fano Inequalities for R(X))**.** *The optimal Fano and reverse-Fano inequalities for the randomness measure R(X) of any M-ary random variable X in terms of $P_{e}=P_{e}\left(X\right)$ are given analytically by the following:*

(59) $$R\left(1\;-\;P_{e},\ldots ,1\;-\;P_{e},1\;-\;\left\lfloor \frac{1}{1-P_{e}}\right\rfloor \left(1\;-\;P_{e}\right),0,\ldots ,0\right)\leq R\left(X\right)\leq R\left(1\;-\;P_{e},\frac{P_{e}}{M-1},\ldots ,\frac{P_{e}}{M-1}\right).$$

**Proof.**  The proof is immediate from Lemma 3 and Theorem 3. The Fano and reverse-Fano bounds are achieved by the distributions on the left and right sides of (58), respectively. □

A similar proof holding for any Schur concave R(X) was already given by Vajda and Vašek [17].

**Figure 2:**
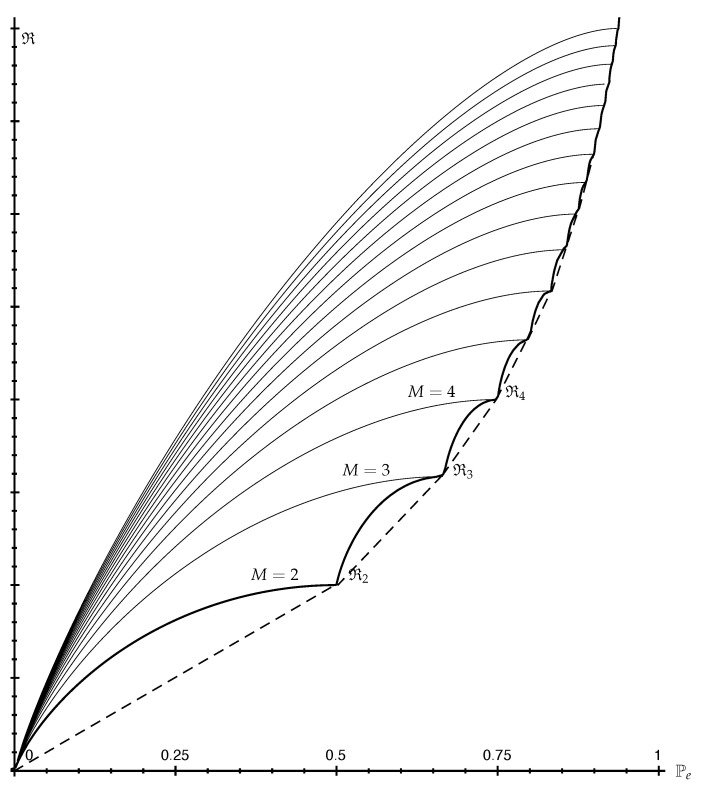
Typical upper Fano bounds (thin) for M=2 to 16 and lower reverse-Fano bound for R(X) (solid) and for R(X|Y) (dashed).

Assuming the zero randomness convention for simplicity (Remark 14), Fano and reverse-Fano bounds can be qualitatively described as follows. They are illustrated in Figure 2.

**Proposition 1** (Shape of Fano Bounds)**.** *The (upper) Fano bound:*

(60) $$P_{e}\in \left[0,1\;-\;\frac{1}{M}\right]\mapsto R\left(1\;-\;P_{e},\frac{P_{e}}{M-1},\ldots ,\frac{P_{e}}{M-1}\right)\in \left[0,R_{M}\right]$$

*where $R_{M}$ denotes maximal randomness (Remark 15) is continuous in $P_{e}>0$, concave in $P_{e}$ and increases from* 0 *(for $P_{e}=0$) to $R_{M}$ (for $P_{e}=1\;-\;\frac{1}{M}$). For any fixed $P_{e}$, it also increases with M.*

**Proof.**  Since R(p)≥0 is concave over $P_{M}$ (Theorem 2), it is continuous on the interior of $P_{M}$. Since $P_{e}\mapsto \left(1\;-\;P_{e},\frac{P_{e}}{M-1},\ldots ,\frac{P_{e}}{M-1}\right)$ is linear, the Fano bound results from the composition of a linear and a concave function. It is, therefore, concave, and continuous at every $P_{e}>0$. It is clear from Lemma 3, or using a suitable Robin Hood operation, that the maximizing distribution becomes more equal as $P_{e}$ increases. Therefore, the Fano bound increases with $P_{e}$. The maximum is attained for $P_{e}=1-\frac{1}{M}$, which corresponds to the uniform distribution achieving maximum randomness $R_{M}$. For fixed $P_{e}$, it is also clear, using a suitable Robin Hood operation, that the maximizing distribution becomes more equal if *M* is increased by one. Therefore, the Fano bound also increases with *M*. □

**Proposition 2** (Shape of reverse-Fano Bounds)**.** *The (lower) reverse-Fano bound:*

(61) $$P_{e}\in \left[0,1\;-\;\frac{1}{M}\right]\mapsto R\left(1\;-\;P_{e},\ldots ,1\;-\;P_{e},1\;-\;\left\lfloor \frac{1}{1-P_{e}}\right\rfloor \left(1\;-\;P_{e}\right),0,\ldots ,0\right)\in \left[0,R_{M}\right]$$

*is continuous in $P_{e}>0$, increases from* 0 *(for $P_{e}=0$) to $R_{M}$ (for $P_{e}=1\;-\;\frac{1}{M}$) and is composed of continuous concave increasing curves connecting successive points ($P_{e}=1-\frac{1}{k}$, $R=R_{k}$) for k=1,2,…,M.*

**Proof.**  For any k∈{1,2,…,M}, the reverse-Fano bound at $P_{e}=1-\frac{1}{k}$ is $R\left(\frac{1}{k},\ldots ,\frac{1}{k}\right)=R_{k}$. It suffices to prove that the reverse-Fano bound is continuous, concave and increasing for $1-\frac{1}{k}\leq P_{e}\leq 1-\frac{1}{k+1}$. When $\lfloor \frac{1}{1-P_{e}}\rfloor =k$, that is, $1-\frac{1}{k}\leq P_{e}<1-\frac{1}{k+1}$, the reverse-Fano bound is $R(1\;-\;P_{e},\ldots ,1\;-\;P_{e},1\;-\;k(1\;-\;P_{e}))$. This results from the composition of a linear and a concave function R(p), which is continuous in the interior of $P_{k}$. Therefore, it is concave in $P_{e}$, and continuous on the whole closed interval $\left[1-\frac{1}{k},1-\frac{1}{k+1}\right]$. Finally, it is clear from Lemma 2 or using a suitable Robin Hood operation that $\left(1\;-\;P_{e},\ldots ,1\;-\;P_{e},1\;-\;k(1\;-\;P_{e})\right)$ becomes more equal as $P_{e}$ increases. Therefore, each curve increases from $R_{k}$ to $R_{k+1}$. □

**Remark 17** (Independence of the reverse-Fano Bound from the Alphabet Size)**.** *Contrary to the (upper) Fano bound, the (lower) reverse-Fano bound is achieved by a probability distribution that does not depend on M. As a result, when the definition of R does not itself explicitly depend on M (as is the case for H, $H_{\alpha }$, G, $G_{\rho }$, $P_{e}$, $P_{e}^{m}$, $R_{2}$), the reverse-Fano bound is the same for all M, except that it is truncated up to $P_{e}=1-\frac{1}{M}$, at which point it meets the (upper) Fano bound (see Figure 2).*

**Theorem** **5** (Optimal Fano and Reverse-Fano Inequalities for R(X|Y))**.** *The optimal Fano and reverse-Fano inequalities for the randomness measure R(X|Y) of any M-ary random variable X in terms of $P_{e}=P_{e}\left(X|Y\right)$ are given analytically by the following:*

(62) $$\left(↾\;\frac{1}{P_{s}}\;↿\;P_{s}-1\right)\left\lfloor \frac{1}{P_{s}}\right\rfloor R_{\left\lfloor \frac{1}{P_{s}}\right\rfloor }+\left(1-\left\lfloor \frac{1}{P_{s}}\right\rfloor P_{s}\right)\;↾\;\frac{1}{P_{s}}\;↿\;R_{↾\frac{1}{P_{s}}↿}\leq R\left(X|Y\right)\leq R\left(1\;-\;P_{e},\frac{P_{e}}{M-1},\ldots ,\frac{P_{e}}{M-1}\right).$$

*where we have noted ↾x↿=⌊x⌋+1 (↾x↿ is the usual ceil function ⌈x⌉, unless x is an integer), $P_{s}=1-P_{e}$ and $R_{k}=R\left(\frac{1}{k},\ldots ,\frac{1}{k}\right)$.*

**Proof.**  The Fano region for X|Y=y, i.e., the locus of the points $\left(P_{e}\left(p_{X|y}\right),R\left(p_{X|y}\right)\right)$ for each Y=y, is given by the inequalities (59). From the definition of conditional randomness, the exact locus of points $\left(P_{e}\left(X|Y\right),R\left(X|Y\right)\right)=E_{y}\left(P_{e}\left(p_{X|y}\right),R\left(p_{X|y}\right)\right)$ is composed of all convex combinations of points in the Fano region, that is, its convex envelope. The extreme points $\left(P_{e}=0,R=R_{1}=0\right)$ and $\left(P_{e}=1-\frac{1}{M},R=R_{M}\right)$ are unchanged. The upper Fano bound joining these two extreme points is concave by Proposition 1 and, therefore, already belongs to the convex envelope. It follows that the upper Fano bound in (59) remains the same, as given in (62). However, the lower reverse-Fano bound for R(X|Y) is the convex hull of the lower bound in (59). By Proposition 2, it is easily seen to be the piecewise linear curve joining all singular points ($P_{e}=1-\frac{1}{k}$, $R=R_{k}$) for k=1,2,…,M (see Figure 2). A closed-form expression is obtained by noting that, when $\lfloor \frac{1}{1-P_{e}}\rfloor =k$, that is, $1-\frac{1}{k}\leq P_{e}<1-\frac{1}{k+1}$, the equation of the straight line joining ($1-\frac{1}{k}$, $R_{k}$) and ($1-\frac{1}{k+1}$, $R_{k+1}$) is $\left(\left(k+1\right)P_{s}-1\right)kR_{k}+\left(1-kP_{s}\right)\left(k+1\right)R_{k+1}$. Plugging $k=\lfloor \frac{1}{P_{s}}\rfloor$ and $k+1=↾\;\frac{1}{P_{s}}\;↿$ gives the lower reverse-Fano bound in (62). □

**Remark 18** (Shape of Fano and reverse-Fano bounds for Conditional Randomness)**.** *By Theorem 5, the Fano inequality for the conditional version R(X|Y) takes the same form as for R(X). In particular, it is increasing and concave in $P_{e}\left(X|Y\right)$. Compared to that for R(X), the reverse-Fano bound for R(X|Y), however, is a piecewise linear convex hull. Clearly, it is still continuous and increasing in $P_{e}\left(X|Y\right)$, as illustrated in Figure 2. If the corresponding sequence of slopes $k\left(k+1\right)\left(R_{k+1}-R_{k}\right)$ is increasing in k, then the reverse-Fano bound for R(X|Y) is also convex in $P_{e}\left(X|Y\right)$.*

**Remark** **19** (φ-Fano Bounds)**.** *If φ(R) is used instead of R, where φ is an increasing function (in particular, to define conditional randomness as in Remark 4), then Theorem 4 and the (upper) Fano bound of Theorem 5 can be directly applied to R. When φ is nonlinear, this may result in (upper) Fano bounds that are no longer concave.*

*However, to obtain the reverse-Fano inequalities for R(X|Y), one has to apply Theorem 5 to φ(R(X|Y)) and then apply the inverse function $\varphi^{-1}$ to the left side of (62). When φ is nonlinear, the resulting “reverse-Fano bound” for R(X|Y) will not be piecewise linear anymore. This is the case, e.g., for conditional α-entropies (see Example 15 below).*

**Example 14** (Fano and reverse-Fano Inequalities for Entropy)**.** *For the Shannon entropy, the optimal Fano inequality (right sides of (59) and (62)) takes the form:*

(63) $$\begin{array}{cc} H(X) & \leq h\left(P_{e}\left(X\right)\right)+P_{e}\left(X\right)\;\log\left(M-1\right) \end{array}$$

(64) $$\begin{array}{cc} H(X|Y) & \leq h\left(P_{e}\left(X|Y\right)\right)+P_{e}\left(X|Y\right)\;\log\left(M-1\right) \end{array}$$

*where $h\left(P_{e}\right)=P_{e}\log\frac{1}{P_{e}}+\left(1-P_{e}\right)\log\frac{1}{1-P_{e}}$ is the binary entropy function. Inequality (64) is the original Fano inequality established in 1952 [22], which has become ubiquitous in information theory and in statistics to relate equivocation to probability of error. Inequality (63) trivially follows, in case of blind estimation (Y≡0). That these inequalities are sharp is well known (see, e.g., [32]).*

*The optimal reverse-Fano inequality (left sides of (59) and (62) with $R_{k}=\log k$) takes the form:*

(65) $$\begin{array}{cc} H(X) & \geq \phi \left(P_{s}\left(X\right)\right)=\phi \left(1-P_{e}\left(X\right)\right) \end{array}$$

(66) $$\begin{array}{cc} H(X|Y) & \geq \bar{\phi }\left(P_{s}\left(X|Y\right)\right)=\bar{\phi }\left(1-P_{e}\left(X|Y\right)\right) \end{array}$$

*where*

(67) $$\begin{array}{cc} \phi (x) & =h\left(\lfloor \frac{1}{x}\rfloor x\right)+\left\lfloor \frac{1}{x}\right\rfloor x\log\left\lfloor \frac{1}{x}\right\rfloor \end{array}$$

(68) $$\begin{array}{cc} \bar{\phi }\left(x\right) & =\left(↾\;\frac{1}{x}\;↿\;x-1\right)\left\lfloor \frac{1}{x}\right\rfloor \log\left\lfloor \frac{1}{x}\right\rfloor +\left(1-\lfloor \frac{1}{x}\rfloor x\right)↾\;\frac{1}{x}\;↿\;\log\;↾\;\frac{1}{x}\;↿ \end{array}$$

*These two lower bounds were first derived by Kovalevsky [33] in 1965. Optimality was already proven in [32].*

**Example** **15** (Fano and reverse-Fano Inequalities for α-Entropy)**.** *By Remark 19, the optimal Fano inequality for $H_{\alpha }\left(X\right)$ is obtained as the right side of (59), which gives the following:*

(69) $$H_{\alpha }\left(X\right)\leq \frac{1}{1-\alpha }\log\left({\left(M-1\right)}^{1-\alpha }P_{e}{\left(X\right)}^{\alpha }+P_{s}{\left(X\right)}^{\alpha }\right).$$

*This was proven by Toussaint [34] for 0<α<1 and, independently, by Ben-Bassat and Raviv [35] for α≠1.*

*Additionally, by Remark 19, the optimal Fano inequality for $H_{\alpha }\left(X|Y\right)$ is obtained by averaging over Y the Fano upper bound of $\varphi_{\alpha }\left(H_{\alpha }\left(X|y\right)\right)$, which is of the form $\phi (P_{e}\left(X|y\right))$, where $\phi \left(x\right)=\mathrm{sgn}\left(1-\alpha \right){\left({\left(M-1\right)}^{1-\alpha }x^{\alpha }+{\left(1-x\right)}^{\alpha }\right)}^{1/\alpha }$, which is concave Lemma 1 in [36]. Therefore, the optimal Fano inequality for $H_{\alpha }\left(X|Y\right)$ is likewise obtained as the right side of (62), which gives the following:*

(70) $$H_{\alpha }\left(X|Y\right)\leq \frac{1}{1-\alpha }\log\left({\left(M-1\right)}^{1-\alpha }P_{e}{\left(X|Y\right)}^{\alpha }+P_{s}{\left(X|Y\right)}^{\alpha }\right).$$

*The optimal reverse-Fano inequality for $H_{\alpha }\left(X\right)$ is obtained as the left side of (59). By Remark 19, $H_{\alpha }\left(X|Y\right)$ is obtained by applying $\varphi_{\alpha }^{-1}\left(x\right)=\frac{\alpha }{1-\alpha }\log\left(\mathrm{sgn}\left(1-\alpha \right)x\right)$ to the left side of (62) for $\varphi_{\alpha }\left(H_{\alpha }\left(X|Y\right)\right)$, where $\varphi_{\alpha }$ is given by (30). This gives the following:*

(71) $$\begin{array}{cc} H_{\alpha }\left(X\right) & \geq \phi_{\alpha }\left(P_{s}\left(X\right)\right)=\phi_{\alpha }\left(1-P_{e}\left(X\right)\right) \end{array}$$

(72) $$\begin{array}{cc} H_{\alpha }\left(X|Y\right) & \geq {\bar{\phi }}_{\alpha }\left(P_{s}\left(X|Y\right)\right)={\bar{\phi }}_{\alpha }\left(1-P_{e}\left(X|Y\right)\right) \end{array}$$

*where*

(73) $$\begin{array}{cc} \phi_{\alpha }\left(x\right) & =\frac{1}{1-\alpha }\log\left(\left\lfloor \frac{1}{x}\right\rfloor x^{\alpha }+{\left(1-\left\lfloor \frac{1}{x}\right\rfloor x\right)}^{\alpha }\right) \end{array}$$

(74) $$\begin{array}{cc} {\bar{\phi }}_{\alpha }\left(x\right) & =\frac{\alpha }{1-\alpha }\log\left(\left(↾\;\frac{1}{x}\;↿\;x-1\right){\left\lfloor \frac{1}{x}\right\rfloor }^{\frac{1}{\alpha }}+\left(1-\lfloor \frac{1}{x}\rfloor x\right)\;↾\;\frac{1}{x}\;{↿}^{\frac{1}{\alpha }}\right) \end{array}$$

*Fano and reverse-Fano inequalities for $H_{\alpha }\left(X\right)$ and $H_{\alpha }\left(X|Y\right)$ were recently established by Sason and Verdú [36].*

**Example** **16** (Fano and reverse-Fano Inequalities for non collision $R_{2}$)**.** *Theorem 4 readily gives the optimal Fano region for $R_{2}\left(X\right)$:*

(75) $$1-\left\lfloor \frac{1}{P_{s}}\right\rfloor P_{s}^{2}-{\left(1-\left\lfloor \frac{1}{P_{s}}\right\rfloor P_{s}\right)}^{2}\leq R_{2}\left(X\right)\leq 1-P_{s}^{2}\left(X\right)-\frac{P_{e}^{2}\left(X\right)}{M-1}.$$

*This can also be easily deduced from (69) and (71) for α=2 via (4). Fano and reverse-Fano inequalities for $R_{2}\left(X\right)$ were first stated without proof in [7].*

*The optimal Fano region for $R_{2}\left(X|Y\right)$, however, cannot be directly deduced from that of $H_{2}\left(X|Y\right)$, because a different kind of average over Y is involved. However, a direct application of Theorem 5 with $R_{k}=1-\frac{1}{k}$ gives the optimal Fano region:*

(76) $$P_{e}\left(X|Y\right)\leq R_{2}\left(X|Y\right)\leq 1-P_{s}^{2}\left(X|Y\right)-\frac{P_{e}^{2}\left(X|Y\right)}{M-1}.$$

**Figure 3:**
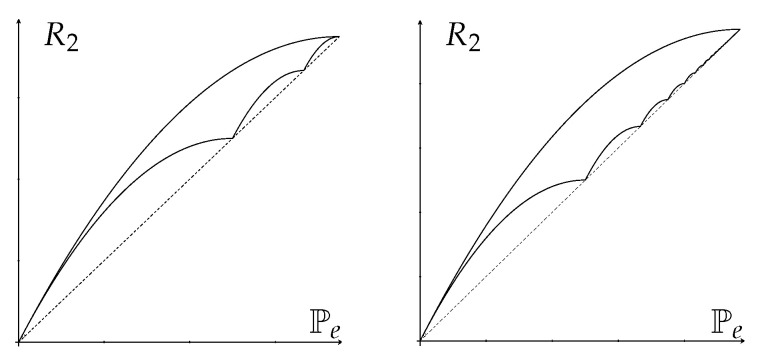
Optimal Fano regions for $R_{2}$ vs. $P_{e}$. Solid: Fano region $R_{2}\left(X\right)$ vs. $P_{e}\left(X\right)$. Dashed: Fano region $R_{2}\left(X|Y\right)$ vs. $P_{e}\left(X|Y\right)$. **Left** M=4; **right** M=32.

*Remarkably, the reverse-Fano inequality has a very simple form $R_{2}\left(X|Y\right)\geq P_{e}\left(X|Y\right)$ (see Figure 3).*

**Example 17** (Fano and reverse-Fano Inequalities for Guessing Entropy)**.** *For guessing entropy G, the Fano inequality is written as shown:*

(77) $$\begin{array}{cc} G(X) & \leq 1+\frac{M}{2}P_{e}\left(X\right) \end{array}$$

(78) $$\begin{array}{cc} G(X|Y) & \leq 1+\frac{M}{2}P_{e}\left(X|Y\right) \end{array}$$

*One obtains similarly $G_{2}\leq 1+\frac{M}{3}\left(M+\frac{5}{2}\right)P_{e}$, $G_{3}\leq 1+\frac{M}{4}\left(M^{2}+3M+4\right)P_{e}$, etc.*

*Due to the fact that $G_{\rho }\left(p\right)$ is linear in p, for fixed $\lfloor \frac{1}{1-P_{e}}\rfloor =k$, the reverse-Fano bound for $G_{\rho }\left(X\right)$ is linear in $P_{e}$. It follows that the bound is already piecewise linear, with a sequence of slopes $s_{k}=k\left(k+1\right)\left(R_{k+1}-R_{k}\right)=k\left(1^{\rho }+\cdots +{\left(k+1\right)}^{\rho }\right)-\left(k+1\right)\left(1^{\rho }+\cdots +k^{\rho }\right)$, which is easily seen to be increasing. Therefore, the (lower) reverse-Fano bound is piecewise linear and convex and coincides with its convex hull. In other words, the reverse-Fano inequality for $G_{\rho }\left(X\right)$ and $G_{\rho }\left(X|Y\right)$ takes the same form:*

(79) $$\begin{array}{cc} G_{\rho }\left(X\right) & \geq \phi_{\rho }\left(P_{s}\left(X\right)\right)=\phi_{\rho }\left(1-P_{e}\left(X\right)\right) \end{array}$$

(80) $$\begin{array}{cc} G_{\rho }\left(X|Y\right) & \geq \phi_{\rho }\left(P_{s}\left(X|Y\right)\right)=\phi_{\rho }\left(1-P_{e}\left(X|Y\right)\right). \end{array}$$

*The following is easily determined from the left side of either (59) or (62):*

(81) $$\phi_{\rho }\left(x\right)=x\left(1^{\rho }+\cdots +{\left\lfloor \frac{1}{x}\right\rfloor }^{\rho }\right)+\left(1-\left\lfloor \frac{1}{x}\right\rfloor x\right){\left\lceil \frac{1}{x}\right\rceil }^{\rho }.$$

*For example, $\phi_{1}\left(x\right)=\left(\left\lfloor \frac{1}{x}\right\rfloor +1\right)\left(1-\left\lfloor \frac{1}{x}\right\rfloor \frac{x}{2}\right)$, such that the following occurs:*

(82) $$\begin{array}{cc} G(X) & \geq \left(\left\lfloor \frac{1}{P_{s}\left(X\right)}\right\rfloor +1\right)\left(1-\left\lfloor \frac{1}{P_{s}\left(X\right)}\right\rfloor \frac{P_{s}\left(X\right)}{2}\right) \end{array}$$

(83) $$\begin{array}{cc} G(X|Y) & \geq \left(\left\lfloor \frac{1}{P_{s}\left(X|Y\right)}\right\rfloor +1\right)\left(1-\left\lfloor \frac{1}{P_{s}\left(X|Y\right)}\right\rfloor \frac{P_{s}\left(X|Y\right)}{2}\right). \end{array}$$

*Fano and reverse-Fano inequalities for $G_{\rho }\left(X|Y\right)$ were recently established by Sason and Verdú [37]. As already shown in [27] for ρ=1, the use of Schur concavity greatly simplifies the derivation.*

**Figure 4:**
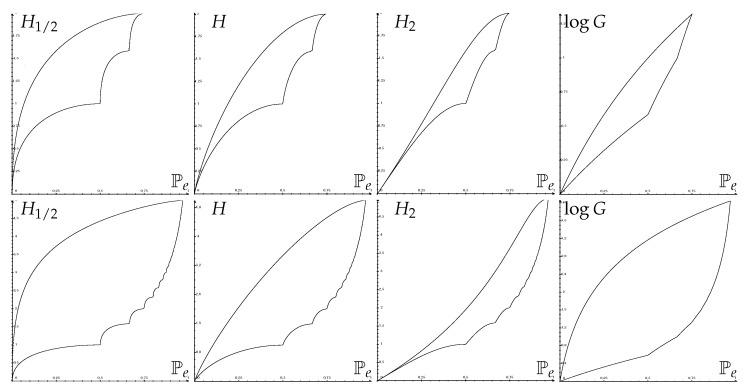
Optimal Fano regions: Entropies (in bits) vs. error probability. **Top** row M=4; **bottom** row M=32.

Figure 4 shows some optimal Fano regions for $H_{1/2}\left(X\right)$, H(X), $H_{2}\left(X\right)$ and logG(X).

## 4. Pinsker and Reverse-Pinsker Inequalities

Pinsker and reverse-Pinsker inequalities relate some divergence measure (e.g., d(p∥q) or $d_{\alpha }\left(p∥q\right)$) between two distributions to their statistical distance Δ(p,q). For simplicity, even though we restrict ourselves to the divergence or distance to the uniform distribution q=u, we still use the generic name “Pinsker inequalities”. Following the discussion in Section 1.6, we adopt the following.

**Definition 7** (Pinsker-type inequalities)**.** *A “Pinsker inequality” (resp. “reverse-Pinsker inequality”) for R(X) gives an upper (resp. lower) bound of R(X) as a function of the statistical randomness R(X) (or statistical distance Δ(p,u)). Pinsker and reverse-Pinsker inequalities are similarly defined for conditional randomness R(X|Y), lower or upper bounded as a function of R(X|Y).*

In this Section, we establish optimal Pinsker and reverse-Pinsker inequalities, where upper and lower bounds are tight. In other words, we determine the maximum and minimum of R for fixed R (or fixed Δ). The exact locus of the region $p\in P_{M}\mapsto \left(R\left(p\right),R\left(p\right)\right)=\left(R\left(X\right),R\left(X\right)\right)$, as well as the exact locus of all attainable values of (R(X|Y),R(X|Y)) is determined analytically for fixed *M*, based on the following.

**Lemma** **4.** *Let R=R(p) and $\Delta =\Delta \left(p,u\right)=1-\frac{1}{M}-R$. For any M-ary probability distribution $p\in P_{M}$ and any integer K such that*

(84) $$|\left\{p>\frac{1}{M}\right\}\left|\leq K\leq \right|\left\{p\geq \frac{1}{M}\right\}|,$$

*where |A| denotes the cardinality of the set A, one has the following:*

(85) $$\left(\Delta +\frac{1}{M},\underset{\left\lfloor MR\right\rfloor \;times}{\underbrace{\frac{1}{M},\ldots ,\frac{1}{M}}},R\;-\;\frac{\left\lfloor MR\right\rfloor }{M},0,\ldots ,0\right)⪯p⪯\left(\underset{K\;times}{\underbrace{\frac{1}{M}\;+\;\frac{\Delta }{K},\ldots ,\frac{1}{M}\;+\;\frac{\Delta }{K}}},\underset{M\;-\;K\;times}{\underbrace{\frac{1}{M}\;-\;\frac{\Delta }{M-K},\ldots ,\frac{1}{M}\;-\;\frac{\Delta }{M-K}}}\right).$$

**Proof.**  Let $T_{+}$ be defined as in (17) for a uniform distribution q=u. Then, $K=|T_{+}|$ satisfies (84), and (16) gives $\Delta =P\left(T_{+}\right)-\frac{K}{M}$. First, consider the largest *K* probabilities, which are all $\geq \frac{1}{M}$ and sum to $P\left(T_{+}\right)=\frac{K}{M}+\Delta$. One obtains the following:

(86) $$\frac{1}{M}+\left(\Delta ,0,\ldots ,0\right)⪯\left(p_{\left(1\right)},p_{\left(2\right)},\ldots ,p_{\left(K\right)}\right)⪯\left(\frac{1}{M}\;+\;\frac{\Delta }{K},\ldots ,\frac{1}{M}\;+\;\frac{\Delta }{K}\right)$$

where, on the right side, we have used Lemma 1 and, on the left side, we have used Lemma 2, applied to $\left(p_{\left(1\right)}-\frac{1}{M},p_{\left(2\right)}-\frac{1}{M},\ldots ,p_{\left(K\right)}-\frac{1}{M}\right)$, which sum to Δ. Next, consider the smallest M−K probabilities, which are all $\leq \frac{1}{M}$ and sum to $1-P\left(T_{+}\right)=\frac{M-K}{M}-\Delta$. One has the following:

(87) $$\left(\frac{1}{M},\ldots ,\frac{1}{M},r,0,\ldots ,0\right)⪯\left(p_{\left(K+1\right)},p_{\left(K+2\right)},\ldots ,p_{\left(M\right)}\right)⪯\left(\frac{1}{M}\;-\;\frac{\Delta }{M-K},\ldots ,\frac{1}{M}\;-\;\frac{\Delta }{M-K}\right)$$

where, on the right side, we have used Lemma 1 and, on the left side, we have used Lemma 2 with $P=\frac{1}{M}$. Combining (86) and (87) gives (85), where the remainder component $0\leq r<\frac{1}{M}$ is computed so that the sum of probabilities on the left side equals one, which gives $r=\left(1-\Delta \right)-\frac{\left\lfloor M(1-\Delta )\right\rfloor }{M}=R-\frac{\left\lfloor MR\right\rfloor }{M}$. □

**Theorem** **6** (Optimal Pinsker and Reverse-Pinsker Inequalities for R(X))**.** *The optimal Pinsker and reverse-Pinsker inequalities for the randomness measure R(X) of any M-ary random variable X in terms of R=R(X) are given analytically as below:*

(88) $$R\left(1-R,\frac{1}{M},\ldots ,\frac{1}{M},R\;-\;\frac{\left\lfloor MR\right\rfloor }{M},0\ldots \right)\;\leq \;R\left(X\right)\;\leq \;\max_{K}R\left(\frac{1}{M}\;+\;\frac{\Delta }{K},\ldots ,\frac{1}{M}\;+\;\frac{\Delta }{K},\frac{1}{M}\;-\;\frac{\Delta }{M-K},\ldots ,\frac{1}{M}\;-\;\frac{\Delta }{M-K}\right)$$

*where $\Delta =1\;-\;\frac{1}{M}\;-\;R$ and the maximum is over all integers 1≤K≤⌊M(1−Δ)⌋=1+⌊MR⌋.*

**Proof.**  Apply Lemma 4 and Theorem 3. The Pinsker and reverse-Pinsker bounds are achieved by the distributions on the left and right sides of (85), respectively. The best value of *K* maximize the randomness R of the distribution on the right side of (85), with the constraint $\frac{1}{M}\;-\;\frac{\Delta }{M-K}\geq 0$, that is, K≤M(1−Δ). □

**Figure 5:**
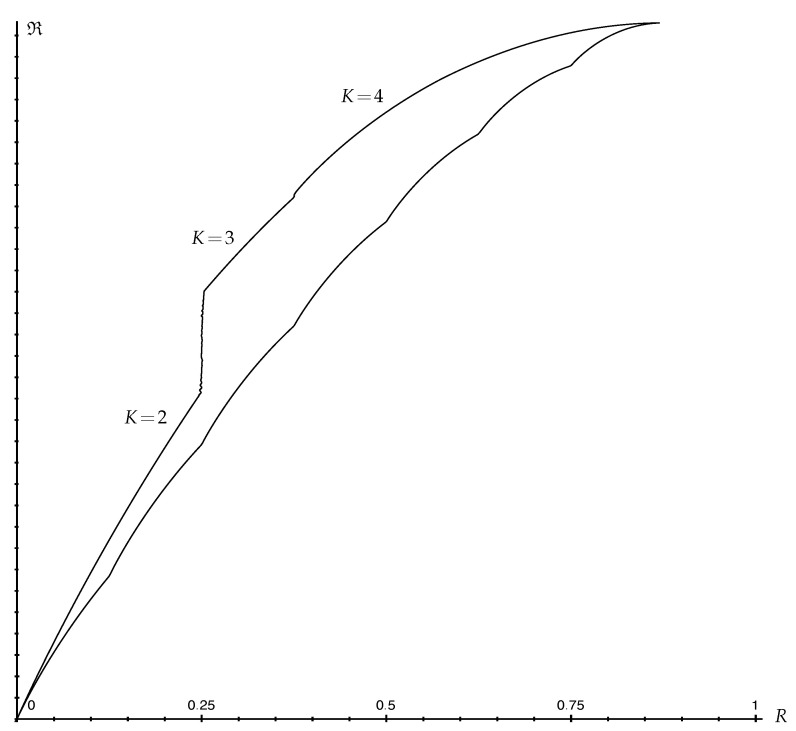
Typical lower and upper Pinsker bounds for M=8. Some optimal values of *K* are given in this example.

Assuming the zero randomness convention for simplicity (Remark 14), Pinsker and reverse-Pinsker bounds can be qualitatively described as follows. They are illustrated in Figure 5.

**Proposition 3** (Shape of Pinsker Bounds)**.** *The (upper) Pinsker bound:*

(89) $$R\in \left[0,1-\frac{1}{M}\right]\mapsto \max_{K}R\left(\frac{1}{M}\;+\;\frac{\Delta }{K},\ldots ,\frac{1}{M}\;+\;\frac{\Delta }{K},\frac{1}{M}\;-\;\frac{\Delta }{M-K},\ldots ,\frac{1}{M}\;-\;\frac{\Delta }{M-K}\right)\in \left[0,R_{M}\right]$$

*where $\Delta =1\;-\;\frac{1}{M}\;-\;R$ and the maximum is over all integers 1≤K≤⌊M(1−Δ)⌋=1+⌊MR⌋, is increasing and piecewise continuous in each subinterval $\left[\frac{k}{M},\frac{k+1}{M}\right]$, (k=0,…,M−1), with possible jump discontinuities at points $\frac{k}{M}$ (k=1,…,M−2).*

**Proof.**  First, notice that the distributions $\left(\frac{1}{M}\;+\;\frac{\Delta }{K},\ldots ,\frac{1}{M}\;+\;\frac{\Delta }{K},\frac{1}{M}\;-\;\frac{\Delta }{M-K},\ldots ,\frac{1}{M}\;-\;\frac{\Delta }{M-K}\right)$ are not necessarily comparable in terms of equalization (partial) order for different values of *K*. It follows that, in general, the optimal value of *K* maximizing $R(\frac{1}{M}\;+\;\frac{\Delta }{K},\ldots ,\frac{1}{M}\;+\;\frac{\Delta }{K},\frac{1}{M}\;-\;\frac{\Delta }{M-K},\ldots ,\frac{1}{M}\;-\;\frac{\Delta }{M-K})$ depends not only on Δ (or *R*), but also on the choice of the randomness measure R.

However, for fixed *K*, $\Delta \mapsto (\frac{1}{M}\;+\;\frac{\Delta }{K},\ldots ,\frac{1}{M}\;+\;\frac{\Delta }{K},\frac{1}{M}\;-\;\frac{\Delta }{M-K},\ldots ,\frac{1}{M}\;-\;\frac{\Delta }{M-K})$ is linear. In addition, since R(p)≥0 is concave over $P_{M}$ (Theorem 2), it is continuous on the interior of $P_{M}$. Therefore, the bound $R(\frac{1}{M}\;+\;\frac{\Delta }{K},\ldots ,\frac{1}{M}\;+\;\frac{\Delta }{K},\frac{1}{M}\;-\;\frac{\Delta }{M-K},\ldots ,\frac{1}{M}\;-\;\frac{\Delta }{M-K})$ results from the composition of a linear and a continuous concave function. It is, therefore, continuous and concave over the domain K≤1+⌊MR⌋, that is, $R\in [\frac{K-1}{M},1-\frac{1}{M}]$. Also, it is clear, using a suitable Robin Hood operation, that, for a fixed *K*, $R(\frac{1}{M}\;+\;\frac{\Delta }{K},\ldots ,\frac{1}{M}\;+\;\frac{\Delta }{K},\frac{1}{M}\;-\;\frac{\Delta }{M-K},\ldots ,\frac{1}{M}\;-\;\frac{\Delta }{M-K})$ is decreasing in Δ, and, therefore, increasing in *R*.

It follows that the (upper) Pinsker bound is a maximum of at most M increasing continuous concave functions, defined over intervals of the form $\left[\frac{K-1}{M},1-\frac{1}{M}\right]$. It is, therefore, increasing over the entire interval $\left[0,1-\frac{1}{M}\right]$ and piecewise continuous in each subinterval $\left[\frac{k}{M},\frac{k+1}{M}\right]$, with possible jumps at the endpoints (see Figure 5). □

**Proposition 4** (Shape of reverse-Pinsker Bounds)**.** *The (lower) reverse-Pinsker bound:*

(90) $$R\in \left[0,1-\frac{1}{M}\right]\mapsto R\left(1-R,\frac{1}{M},\ldots ,\frac{1}{M},R\;-\;\frac{\left\lfloor MR\right\rfloor }{M},0\ldots \right)\in \left[0,R_{M}\right]$$

*is continuous in R>0, increases from* 0 *(for R=0) to $R_{M}$ (for $R=1\;-\;\frac{1}{M}$) and is composed of continuous concave increasing curves connecting successive points ($R=\frac{k}{M}$, $R=r_{k}$ for k=0,1,…,M−1, where the following holds:*

(91) $$r_{k}=R\left(1-\frac{k}{M},\frac{1}{M},\ldots ,\frac{1}{M}\right).$$

**Proof.**  For fixed k=⌊MR⌋, that is, $\frac{k}{M}\leq R<\frac{k+1}{M}$, the bound $R(1-R,\frac{1}{M},\ldots ,\frac{1}{M},R\;-\;\frac{k}{M},0\ldots )$ results from the composition of a linear and a concave function. It is, therefore, concave, and continuous at every R>0. It is clear, using a suitable Robin Hood operation on $\left(1-R,R\;-\;\frac{k}{M}\right)$, that this bound increases with *R* on the subinterval $\left[\frac{k}{M},\frac{k+1}{M}\right]$. For $R=\frac{k}{M}$, it equals $R\left(1-\frac{k}{M},\frac{1}{M},\ldots ,\frac{1}{M}\right)=r_{k}$, which is easily seen, using a suitable Robin Hood operation, to be increasing with *k*, with maximum $r_{M-1}=R_{M}$. □

**Theorem** **7** (Optimal Pinsker and Reverse-Pinsker Inequalities for R(X|Y))**.** *The optimal Pinsker and reverse-Pinsker inequalities for the randomness measure R(X|Y) of any M-ary random variable X in terms of R=R(X|Y) are given by the convex envelope of the Pinsker region determined by (88). In particular, consider the following:*

- *If the (upper) Pinsker bound for R(X) is concave (with no discontinuities), then the same optimal bound holds for R(X|Y) in terms of $R\left(X|Y\right)=R=1-\frac{1}{M}-\Delta$:*
  (92) $$R\left(X|Y\right)\leq \max_{K}R\left(\frac{1}{M}\;+\;\frac{\Delta }{K},\ldots ,\frac{1}{M}\;+\;\frac{\Delta }{K},\frac{1}{M}\;-\;\frac{\Delta }{M-K},\ldots ,\frac{1}{M}\;-\;\frac{\Delta }{M-K}\right);$$
- *If the sequence $r_{k}-r_{k-1}$ (k=1,…,M−1) is nondecreasing, where $r_{k}$ is defined by (91), then the optimal (lower) reverse-Pinsker bound for R(X|Y) is given by the piecewise linear function connecting points $\left(\frac{k}{M},r_{k}\right)$;*
- *If the sequence $r_{k}-r_{k-1}$ (k=1,…,M−1) is nonincreasing, then the optimal (lower) reverse-Pinsker bound for R(X|Y) writes as follows:*
  (93) $$R\left(X|Y\right)\geq \frac{R_{M}-R_{0}}{1-1/M}R\left(X|Y\right)+R_{0}$$
  *where, as before: $R_{k}=R\left(\frac{1}{k},\ldots ,\frac{1}{k}\right)$ and $R_{0}=R\left(0\right)$.*

**Proof.**  The Pinsker region for X|Y=y, i.e., the locus of the points $\left(R\left(p_{X|y}\right),R\left(p_{X|y}\right)\right)$ for each Y=y, is given by the inequalities (88). From the definition of conditional randomness, the exact locus of points $\left(R\left(X|Y\right),R\left(X|Y\right)\right)=E_{y}\left(R\left(p_{X|y}\right),R\left(p_{X|y}\right)\right)$ is composed of all convex combinations of points in the Pinsker region, that is, its convex envelope.

The extreme points $\left(R=0,R=R_{1}=0\right)$ and $\left(R=1-\frac{1}{M},R=R_{M}\right)$ are unchanged. The upper Pinsker bound joining these two extreme points is piecewise concave by Proposition 3 and, therefore, if continuous, already belongs to the convex envelope. It follows, in this case, that the upper Pinsker bound in (88) remains the same, as given in (92).

The lower reverse-Pinsker bound for R(X|Y) is the convex hull of the lower bound in (88). By Proposition 4, if the sequence $r_{k}-r_{k-1}$ is non nondecreasing, the piecewise linear curve joining all singular points ($R=\frac{k}{M}$, $R=r_{k}$) for k=0,1,…,M−1) is convex and already coincides with its convex hull. If, on the contrary, the sequence $r_{k}-r_{k-1}$ is non nonincreasing, that piecewise linear curve is concave, and its convex hull is simply the straight line joining the extreme endpoints (R=0, $R=r_{0}=R_{1}=0$) and ($R=1-\frac{1}{M}$, $R=R_{M}$), which is given by (93). □

**Remark** **20** (φ-Pinsker Bounds)**.** *If φ(R) is used instead of R, where φ is an increasing function (in particular, to define conditional randomness as in Remark 4), then Theorem 6 can be directly applied to R. When φ is nonlinear, this may result in (upper) Pinsker bounds that are no longer concave.*

*However, to obtain the reverse-Pinsker inequalities for R(X|Y), one has to apply Theorem 7 to φ(R(X|Y)) and then apply the inverse function $\varphi^{-1}$ to (92). When φ is nonlinear, the resulting “reverse-Pinsker bound” for R(X|Y) is no longer piecewise linear. This is the case, e.g., for conditional α-entropies (see Example 19 below).*

**Example 18** (Pinsker and reverse-Pinsker Inequalities for Entropy)**.** *For the Shannon entropy, the optimal Pinsker bounds of Theorem 6 are easily determined as shown:*

(94) $$\begin{array}{c} \left(1-R\right)\log\frac{1}{1-R}+\frac{\left\lfloor MR\right\rfloor }{M}\log M+\left(R\;-\;\frac{\left\lfloor MR\right\rfloor }{M}\right)\log\frac{1}{R\;-\;\frac{\left\lfloor MR\right\rfloor }{M}}\\ \leq H(X)\leq \\ \max_{1\leq K\leq \lfloor M(1-\Delta )\rfloor }\left(\left(\frac{K}{M}+\Delta \right)\log\frac{1}{\frac{1}{M}+\frac{\Delta }{K}}+\left(1-\frac{K}{M}-\Delta \right)\log\frac{1}{\frac{1}{M}-\frac{\Delta }{M-K}}\right) \end{array}$$

*where R=R(X) and $\Delta =1-\frac{1}{M}-R\left(X\right)$. The maximizing value of K depends on the value of* Δ*. The lower bound was proven in implicit form in Thm. 3 in [38], while the upper bound was given in Thm. 26 in [39].*

*Here, (91) is of the form $r_{k}=\phi \left(\frac{k}{M}\right)$, where $\phi \left(x\right)=\left(1-x\right)\log\frac{1}{1-x}+x\log M$ is strictly concave increasing for $0\leq x\leq 1-\frac{1}{M}$. As a consequence, the sequence $r_{k}-r_{k-1}$ is decreasing for k=1,…,M−1, and, by Theorem 7, the optimal reverse-Pinsker inequality for conditional entropy is simply the following:*

(95) $$H\left(X|Y\right)\geq \frac{M\log M}{M-1}R\left(X|Y\right).$$

**Example** **19** (Pinsker and reverse-Pinsker Inequalities for α-Entropy and for $R_{2}$)**.** *By Remark 20, the optimal Pinsker and reverse-Pinsker inequalities (88) for α-entropy $H_{\alpha }\left(X\right)$ are given as below:*

(96) $$\begin{array}{c} \frac{1}{1-\alpha }\log\left({\left(1-R\right)}^{\alpha }+\frac{\left\lfloor MR\right\rfloor }{M^{\alpha }}+{\left(R-\frac{\left\lfloor MR\right\rfloor }{M}\right)}^{\alpha }\right)\\ \leq H_{\alpha }\left(X\right)\leq \\ \max_{1\leq K\leq \lfloor M(1-\Delta )\rfloor }\frac{1}{1-\alpha }\log\left(K{\left(\frac{1}{M}+\frac{\Delta }{K}\right)}^{\alpha }+\left(M-K\right){\left(\frac{1}{M}-\frac{\Delta }{M-K}\right)}^{\alpha }\right) \end{array}$$

*where R=R(X) and $\Delta =1-\frac{1}{M}-R\left(X\right)$. Again, the maximizing value of K depends on the value of* Δ*.*

*For collision entropy (α=2), since $K{\left(\frac{1}{M}+\frac{\Delta }{K}\right)}^{2}+\left(M-K\right){\left(\frac{1}{M}-\frac{\Delta }{M-K}\right)}^{2}=\frac{1}{M}+\frac{M\Delta^{2}}{K(M-K)}$ achieves its minimum when the integer K is closest to $\frac{M}{2}$, the optimal Pinsker and reverse-Pinsker inequalities simplify to the following:*

(97) $$-\log\left({\left(1-R\right)}^{2}+\frac{\left\lfloor MR\right\rfloor }{M^{2}}+{\left(R-\frac{\left\lfloor MR\right\rfloor }{M}\right)}^{2}\right)\leq H_{2}\left(X\right)\leq -\log\left(\frac{1}{M}+\frac{M\Delta^{2}}{K^{*}\left(M-K^{*}\right)}\right)$$

*where $K^{*}=\min\left(\left\lfloor \frac{M}{2}\right\rfloor ,\left\lfloor M\left(1-\Delta \right)\right\rfloor \right)$. In terms of $R_{2}$, the optimal Pinsker and reverse-Pinsker inequalities read as shown:*

(98) $$1-{\left(1-R\right)}^{2}-\frac{\left\lfloor MR\right\rfloor }{M^{2}}-{\left(R-\frac{\left\lfloor MR\right\rfloor }{M}\right)}^{2}\leq R_{2}\left(X\right)\leq 1-\frac{1}{M}-\frac{M\Delta^{2}}{K^{*}\left(M-K^{*}\right)}.$$

*Since $x\left(1-x\right)\leq \frac{1}{4}$, one always has $K\left(M-K\right)\leq K^{*}\left(M-K^{*}\right)\leq \frac{M^{2}}{4}$ (maximum achieved when $K^{*}=\frac{M}{2}$), so that the (upper) Pinsker bound can be further bounded:*

(99) $$\begin{array}{cc} H_{2}\left(X\right) & \leq \log\frac{M}{1+4\Delta^{2}},\\ R_{2}\left(X\right) & \leq 1-\frac{1+4\Delta^{2}}{M} \end{array}$$

*This upper bound was derived by Shoup Thm 8.36 in [8] and was later re-derived in the Lemma in 4 [40]. This, however, is the* optimal *Pinsker bound* only *when $K^{*}=\frac{M}{2}$, that is, when M is even and $\Delta \leq \frac{1}{2}$ (i.e., $R\geq \frac{1}{2}-\frac{1}{M}$).*

*By Remark 20, to obtain the optimal reverse-Pinsker inequality for $H_{2}\left(X|Y\right)$, we consider $\varphi_{2}\left(H_{2}\left(X|Y\right)\right)$, where, from (30), $\varphi_{2}\left(x\right)=-\exp\left(-x/2\right)$ and $\varphi_{2}^{-1}\left(y\right)=-2\log\left(-y\right)$. For this quantity, one has, from (91), $r_{k}=\varphi_{2}\left(-\log\left({\left(1-\frac{k}{M}\right)}^{2}+\frac{k}{M^{2}}\right)\right)$ of the form $r_{k}=\phi \left(\frac{k}{M}\right)$, where $\phi \left(x\right)=-\sqrt{{\left(1-x\right)}^{2}+\frac{x}{M}}$ is strictly concave increasing for $0\leq x\leq 1-\frac{1}{M}$. As a consequence, the sequence $r_{k}-r_{k-1}$ is decreasing for k=1,…,M−1, and, by Theorem 7, the optimal reverse-Pinsker bound for conditional 2-entropy is $\varphi_{2}^{-1}\left(\frac{\varphi_{2}\left(\log M\right)-\varphi_{2}\left(0\right)}{1-1/M}R\left(X|Y\right)+\varphi_{2}\left(0\right)\right)$, which gives the optimal reverse-Pinsker inequality:*

(100) $$H_{2}\left(X|Y\right)\geq -2\log\left(1-\frac{R(X|Y)}{1+\frac{1}{\sqrt{M}}}\right).$$

*For $R_{2}\left(X|Y\right)$, one has $r_{k}=1-{\left(1-\frac{k}{M}\right)}^{2}-\frac{k}{M^{2}}=\psi \left(\frac{k}{M}\right)$, where $\psi \left(x\right)=\left(2-\frac{1}{M}\right)x-x^{2}$ is strictly concave increasing for $0\leq x\leq 1-\frac{1}{M}$. As a consequence, the sequence $r_{k}-r_{k-1}$ is decreasing for k=1,…,M−1, and, since $R_{M}=1-\frac{1}{M}$, by Theorem 7, the optimal reverse-Pinsker inequality for $R_{2}\left(X|Y\right)$ is simply as below:*

(101) $$R_{2}\left(X|Y\right)\geq R\left(X|Y\right)$$

*(see Figure 6).*

**Example 20** (Pinsker and reverse-Pinsker Inequalities for Guessing Entropy)**.** *For the guessing entropy, the optimal Pinsker bounds of Theorem 6 are easily determined:*

(102) $$1+\left(\left\lfloor MR\left(X\right)\right\rfloor +1\right)\left(R\left(X\right)-\frac{\left\lfloor MR(X)\right\rfloor }{2M}\right)\leq G\left(X\right)\leq 1+\frac{MR(X)}{2}.$$

*A notable property is that the optimal upper bound does not depend on the value of K. The upper bound is mentioned by Pliam in [4] as an upper bound of Δ(p,u). The methodology of this paper, based on Schur concavity, greatly simplifies the derivation.*

*For the conditional guessing entropy G(X|Y), observe that the upper Pinsker bound for G(X) is linear (hence, concave) in R and that (91) is of the form $r_{k}=1+\frac{k(k+1)}{2M}$, where the sequence $r_{k}-r_{k-1}=\frac{k}{M}$ is increasing. Therefore, by Theorem 7, the optimal Pinsker region for conditional entropy G(X|Y) is the same as for G(X):*

(103) $$1+\left(\left\lfloor MR\left(X|Y\right)\right\rfloor +1\right)\left(R\left(X|Y\right)-\frac{\left\lfloor MR(X|Y)\right\rfloor }{2M}\right)\leq G\left(X|Y\right)\leq 1+\frac{MR(X|Y)}{2}.$$

**Figure 7:**
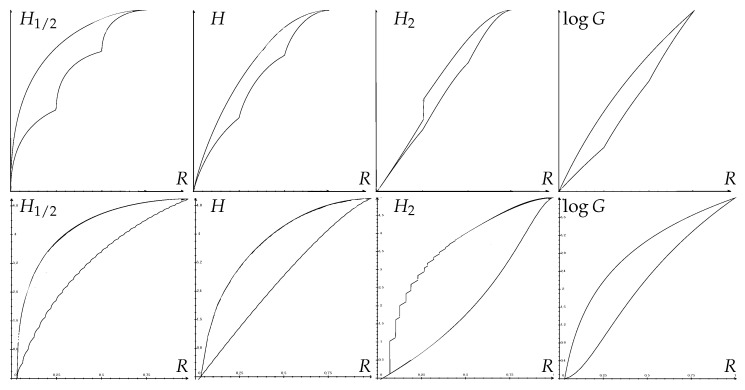
Optimal Pinsker regions: Entropies (in bits) vs. statistical randomness *R*. **Top** row M=4; **bottom** row M=32.

Figure 7 shows some optimal Pinsker regions for $H_{1/2}\left(X\right)$, H(X), $H_{2}\left(X\right)$ and logG(X).

**Example 21** (Statistical Randomness vs. Probability of Error)**.** *As a final example, we present the optimal regions of statistical randomness R vs. probability of error $P_{e}$. In this case, observe the following from Definitions 6 and 7:*

- *The (optimal) Fano inequality for R is the same as the (optimal) reverse-Pinsker inequality for $P_{e}$;*
- *The (optimal) Pinsker inequality for $P_{e}$ is the same as the (optimal) reverse-Fano inequality for R.*

*Letting R=R(X) and $P_{s}=P_{s}\left(X\right)$, Theorem 4 readily gives the optimal Fano and reverse-Fano inequalities:*

(104) $$\frac{1}{2}\left(1-\frac{1}{M}-\left(P_{s}-\frac{2}{M}\right)\left\lfloor \frac{1}{P_{s}}\right\rfloor -\left|1-\left\lfloor \frac{1}{P_{s}}\right\rfloor P_{s}-\frac{1}{M}\right|\right)\leq R\left(X\right)\leq P_{e}\left(X\right)$$

*while Theorem 6 gives the optimal Pinsker and reverse-Pinsker inequalities:*

(105) $$R\left(X\right)\leq P_{e}\left(X\right)\leq 1-\frac{1}{M}-\frac{\Delta }{\left\lfloor M(1-\Delta )\right\rfloor }=\frac{R+\left\lfloor MR\right\rfloor -\frac{\left\lfloor MR\right\rfloor }{M}}{1+\lfloor MR\rfloor }$$

*since the maximum of $1-\frac{1}{M}-\frac{\Delta }{K}$ in the right side of (88) is for maximum K=⌊M(1−Δ)⌋.*

*Similarly, letting R=R(X|Y) and $P_{s}=P_{s}\left(X|Y\right)$, Theorem 5 with $R_{k}=\frac{k-1}{M}$ readily gives the optimal Fano and reverse-Fano inequalities:*

(106) $$\frac{\left(↾\;\frac{1}{P_{s}}\;↿\;P_{s}-1\right)\left({\left\lfloor \frac{1}{P_{s}}\right\rfloor }^{2}\;-\;\left\lfloor \frac{1}{P_{s}}\right\rfloor \right)+\left(1-\left\lfloor \frac{1}{P_{s}}\right\rfloor P_{s}\right)\left({↾\;\frac{1}{P_{s}}\;↿}^{2}\;-\;↾\;\frac{1}{P_{s}}\;↿\right)}{M}\leq R\left(X|Y\right)\leq P_{e}\left(X|Y\right)$$

*while Theorem 7 gives the optimal Pinsker and reverse-Pinsker inequalities:*

(107) $$R\left(X|Y\right)\leq P_{e}\left(X|Y\right)\leq 1-\frac{2}{\lfloor MR\rfloor +2}+\frac{MR}{\left(\lfloor MR\rfloor +1)(\lfloor MR\rfloor +2\right)}$$

*where the upper bound is the piecewise linear function connecting points $\left(P_{e}=1-\frac{1}{k+1},R=\frac{k}{M}\right)$ for k=0,1,…,M−1.*

**Figure 8:**
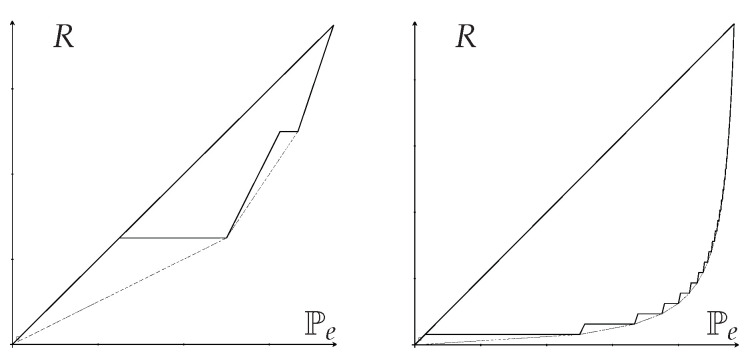
Optimal Fano/Pinsker region for *R* vs. $P_{e}$. Solid: region R(X) vs. $P_{e}\left(X\right)$. Dashed: region R(X|Y) vs. $P_{e}\left(X|Y\right)$. **Left** M=4; **right** M=32.

*From the above observation, the left (reverse-Fano) inequality in (104) is equivalent to the right (Pinsker) inequality in (105), and, similarly, the left (reverse-Fano) inequality in (106) is equivalent to the right (Pinsker) inequality in (107), which do not seem obvious from the expressions above. The optimal Fano/Pinsker region is illustrated in Figure 8.*

## 5. Some Applications

Fano and Pinsker inequalities find many applications in many areas of science; we only mention a few. They have been applied in character recognition [33], feature selection [7], Bayesian statistical experiments [17], statistical data processing [13], quantization [41], hypothesis testing [36], entropy estimation [38], channel coding [42], sequential decoding [11] and list decoding [36,43], lossless compression [37,43,44] and guessing [37,44], knowledge representation [12], cipher security measures [4], hash functions [8], randomness extractors [40], information flow [18], statistical decision making [20] and side-channel analysis [14,27,45]. Some of the various inequalities used for these applications are not optimal (or not proven optimal) for various reasons (simplicity of the expressions, approximations, etc.). By contrast, the methodology of this paper always provides optimal direct or reverse-Fano and -Pinsker inequalities.

## 6. Conclusions and Perspectives

We have derived optimal regions for randomness measures compared to either the error probability or the statistical randomness (or the total variation distance). One perspective is to provide similar optimal regions relating two arbitrary randomness measures. Of course, by (6), Fano regions such as $H_{\alpha }$ vs. $P_{e}$ can be trivially reinterpreted as regions $H_{\alpha }$ vs. $H_{\infty }$ (see, e.g., Figure 2 in [42] for the region H vs. $H_{\infty }$). Using some more involved derivations, the authors of [46] have investigated the optimal regions H vs. $H_{2}$ and, more generally, the authors of [47,48] have investigated the optimal regions between two *α*-entropies of different orders. It would be desirable to apply the methods of this paper to the more general case of two arbitrary randomness measures. In particular, the determination of the optimal regions $H_{\alpha }$ vs. $G_{\rho }$ will allow one to assess the sharpness of the “Massey-type” inequalities of [5].

Catalytic majorization [49] was found to be a necessary and sufficient condition for the increase of all Rényi entropies (including the ones with negative parameters *α*). It would be interesting to find similar necessary and sufficient conditions for other types of randomness measures.

It is also possible to generalize the notion of entropies and other randomness quantities with respect to an arbitrary dominating measure instead of the counting measure, e.g., to extend the considerations of this paper from the discrete case to the continuous case. The relevant notion of majorization in this more general context is studied, e.g., in [50].

Concerning Pinsker regions, another perspective is to extend the results of this paper to the more general case of Pinsker and reverse-Pinsker inequalities, relating “distances” of two arbitrary distributions p,q by removing the restriction that q=u is uniform. Some results in this direction appear in [38,51,52,53,54,55,56,57].

Other types of inequalities on randomness measures with different constraints can also be obtained via majorization theory [43,44].

## Figures and Tables

**Figure 6:**
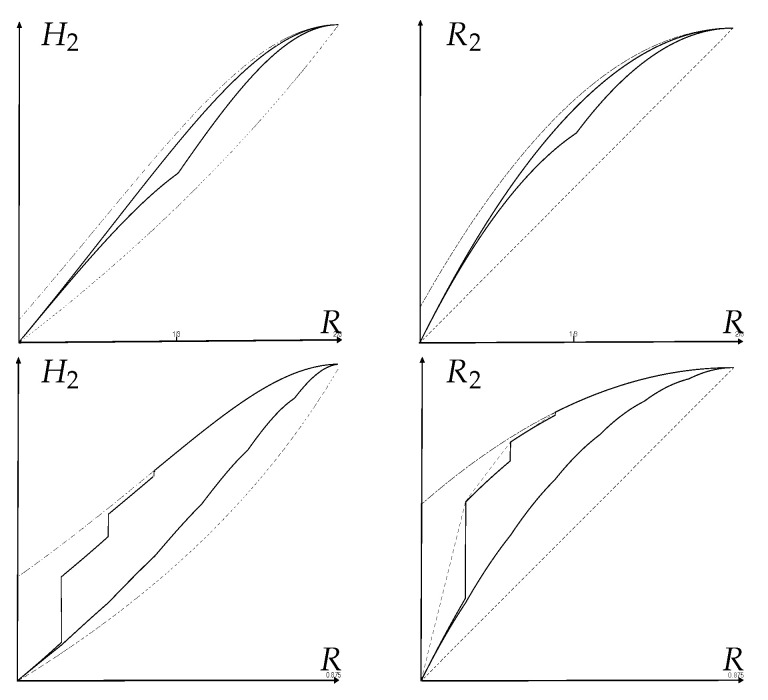
Optimal Pinsker regions: $H_{2}$ (in bits) and $R_{2}$ vs. statistical randomness *R*. Solid: Pinsker region $H_{2}\left(X\right)$ (resp. $R_{2}\left(X\right)$) vs. R(X). Dashed: Pinsker region $H_{2}\left(X|Y\right)$ (resp. $R_{2}\left(X|Y\right)$) vs. R(X|Y). Dash-dotted: Shoup’s upper bound (99). **Top** row M=3; **bottom** row M=8.

## Data Availability

Not applicable.

## Abbreviations

The following abbreviations are used in this manuscript:

X∼p	*X* follows the probability distribution *p*
$H=H_{1}$	Shannon entropy
$H_{2}$	collision entropy
$H_{\infty }$	min-entropy
$H_{\alpha }$	α-entropy
$G=G_{1}$	guessing entropy
$G_{\rho }$	ρ-guessing moment
$P_{e}$	probability of error
$P_{e}^{m}$	error probability of order *m*
$P_{s}=1-P_{e}$	probability of success
$R=R_{1}$	statistical randomness
$\Delta =1-\frac{1}{M}-R$	statistical distance to the uniform
$R_{2}$	complementary index of coincidence
R	any randomness measure
